# AutoKFL: Linux Kernel Fault Localization via ReAct-Based Multi-Agent Framework with Dynamic Crash Reproduction

**DOI:** 10.3390/s26134129

**Published:** 2026-06-30

**Authors:** JungWoo Park, Minju Kang, Seungho Jeon, Seong Oun Hwang

**Affiliations:** 1 Department of Computer Engineering, Gachon University, Seongnam 13120, Republic of Korea; wjddn0623@gachon.ac.kr; 2 Department of Information Security, Gachon University, Seongnam 13120, Republic of Korea; bianka1591@gachon.ac.kr; 3 Department of Smart Security, Gachon University, Seongnam 13120, Republic of Korea

**Keywords:** fault localization, Linux kernel, multi-agent system, large language model

## Abstract

Fault localization (FL) is the task of identifying code locations responsible for bugs in software, and it is a prerequisite step in the bug-fixing process. FL in large-scale systems such as the Linux kernel involves three core challenges: First, the vast codebase fundamentally complicates fault search. Second, the structural characteristics of the kernel environment severely restrict runtime visibility. Third, the diverse and non-trivial root causes of kernel faults expand the reasoning space exponentially. To address these challenges, we make the following observations: (1) decomposing the analysis process into functionally separated agents progressively narrows the search scope, (2) sufficient information for analysis can be extracted from static artifacts collected at crash time without runtime instrumentation, and (3) iterative interaction among agents extends the search scope to non-trivial root causes. Based on these observations, we propose AutoKFL, an automated FL system for Linux kernel crashes. AutoKFL employs four cooperating large language model (LLM)-based agents—crash observer, code collector, code analyzer, and evidence synthesizer—to perform crash observation, code collection, code analysis, and evidence synthesis in sequence. Each agent operates in a reasoning–acting (ReAct) manner and supports iterative exploration through conditional routing that allows returning to a prior stage when necessary. In experiments on 208 Linux kernel crashes reported on Syzbot, AutoKFL achieved a file-level Recall@1 of 0.77 and a mean reciprocal rank (MRR) of 0.822, outperforming single-LLM-call approaches across both file-level and function-level localization.

## 1. Introduction

Fault localization (FL) is the task of identifying code locations responsible for bugs in software, and it constitutes a prerequisite step in the bug-fixing process. According to prior studies, developers spend a significant portion of total debugging time on fault localization [1], motivating a diverse body of research aimed at automating this process. However, existing FL techniques have primarily been developed and evaluated on software of moderate scale, and their applicability to large-scale systems such as the Linux kernel remains insufficiently explored. The Linux kernel comprises tens of thousands of files and tens of millions of lines of code, and kernel faults can cause wide-ranging damage including system crashes, data corruption, and security vulnerabilities. In an empirical study applying state-of-the-art large language model (LLM) agent-based FL techniques to the kernel, file-level Top-1 accuracy reached only 41.6% [2], indicating that FL techniques tailored to the kernel environment are needed.

Automated FL approaches are broadly categorized into coverage-based, information retrieval-based, learning-based, and LLM agent-based methods. Coverage-based approaches, specifically spectrum-based fault localization (SBFL), statistically analyze test execution coverage to estimate suspicious locations [3,4]. Information retrieval-based fault localization (IRFL) ranks faulty files by measuring textual similarity between bug reports and the codebase, with the advantage of not requiring test execution [5,6]. Learning-based fault localization (LBFL) predicts fault locations by learning bug patterns through neural networks [7,8]. More recently, agent-based approaches that leverage LLMs’ code understanding capability have attracted attention; systems such as AutoFL [9] and AgentFL [10] enable an LLM to autonomously explore a codebase through function calls and identify fault locations. However, all of these techniques primarily target general user-level software, and their applicability to environments such as the Linux kernel requires further study.

FL for the Linux kernel involves three core challenges that distinguish it from FL for general software: ❶ The vast codebase fundamentally complicates fault search. As of Linux v7.0, the kernel consists of more than 39 million lines of code [11]. Because faults propagate across multiple files along function call chains or data dependencies, existing agents encounter difficulty pinpointing exact fault locations even after narrowing the search to the directory level [2]. ❷ The structural characteristics of the kernel environment severely restrict runtime observability. The kernel strictly limits runtime instrumentation and logging to minimize overhead, and it operates in a privileged mode isolated from the user space. As a result, bug reports often describe only symptoms without detailed execution information, leaving a significant gap from the actual root cause. ❸ The diverse and non-trivial nature of kernel fault root causes expands the reasoning space exponentially. Kernel faults are influenced by a wide range of external factors such as hardware configuration, system load, and timing; existing agents tend to focus only on the most apparent cause and frequently miss the actual root cause [2].

To overcome these three challenges, we present the following key insights: ① Rather than processing the vast codebase in a single reasoning step, decomposing the analysis into multiple functionally separated agents allows the search scope to be progressively narrowed. Each agent selectively collects only relevant code using tools such as function definition lookup, call graph traversal, and data dependency tracing, enabling precise exploration without unnecessary context consumption. ② Even without runtime instrumentation, sufficient information for analysis can be extracted from static artifacts collected at crash time—such as call stacks, processor context, and crash-reproducing code. A dedicated agent systematically derives structured observations that serve as the starting point for subsequent analysis. ③ Instead of pinpointing the root cause in a single inference, iterative interaction among agents generates and examines multiple hypotheses, extending the search scope to non-trivial causes.

This paper proposes AutoKFL, an automated fault localization system for Linux kernel crashes. AutoKFL targets kernel bugs reported by Syzbot, which continuously runs Syzkaller [12] and collects dynamic information—such as call stacks and central processing unit (CPU) context—by reproducing crashes via QEMU [13]. Using these inputs, AutoKFL localizes faults through four specialized agents designed to emulate the analysis process of human experts. Each agent operates in a reasoning–acting (ReAct) [14] manner, iterating between tool calls and reasoning to perform analysis. The crash observer extracts structured information from crash artifacts as the starting point of analysis. The code collector selectively gathers code required for analysis using tools such as function definition lookup, call graph traversal, and data dependency tracing. The code analyzer derives bug scenarios and suspicious locations from the collected code. The evidence synthesizer integrates the outputs of preceding agents and produces as its final output a confidence-ranked list of fault locations and a root-cause explanation. Inter-agent routing is implemented via conditional edges and supports iterative exploration that can return to a prior stage as needed. Kernel reliability is critical for sensor-driven systems, including embedded devices, IoT platforms, and cyber–physical systems. These systems often rely on Linux-based software stacks for sensing, communication, and data processing. By localizing kernel faults from crash reports and reproduction evidence, AutoKFL can support the maintenance of reliable software infrastructures for sensor-driven applications. In experiments on 208 Linux kernel crashes reported on Syzbot, AutoKFL achieved a file-level Recall@1 of 0.77 and a mean reciprocal rank (MRR) of 0.822, demonstrating consistent performance improvements over single-LLM-call approaches at both the file and function levels. This work makes the following three contributions:

- We propose AutoKFL, an automated fault localization system for Linux kernel crashes. AutoKFL is a multi-agent pipeline consisting of four stages—crash observation, code collection, code analysis, and evidence synthesis—where each agent operates in a ReAct manner and utilizes tools specialized for kernel code exploration.
- We present a novel methodology that combines dynamic crash reproduction via QEMU with LLM-based static code analysis. This methodology compensates for the limited runtime visibility in kernel FL using dynamically collected crash artifacts, and it systematically performs large-scale codebase exploration and complex root cause reasoning through agent decomposition that emulates the analysis process of human experts.
- In experiments on 208 Linux kernel crashes reported on Syzbot, AutoKFL achieves a file-level Recall@1 of 0.77 and MRR of 0.822, and demonstrates consistent performance superiority over single-LLM-call approaches and ablation variants.

The remainder of this paper is organized as follows: Section 2 reviews related work on spectrum-based, information retrieval-based, learning-based, and LLM-based fault localization techniques. Section 3 describes the overall design of AutoKFL and the role and interaction structure of its four agents. Section 4 presents the experimental setup, dataset description, and results addressing three research questions. Section 5 discusses key findings and limitations derived from the experiments. Finally, Section 6 summarizes this work and outlines directions for future research.

## 2. Related Work

### 2.1. Spectrum-Based Fault Localization

SBFL is a technique that ranks code elements likely to contain faults by statistically analyzing coverage information collected during test execution. Aeneas [15] notes that coverage matrices of real programs are high-dimensional and suffer from severe class imbalance, proposing a universal data augmentation framework that combines dimensionality reduction with a conditional variational autoencoder (CVAE) [16] to generate synthetic failing tests. While Aeneas can be applied independently of existing FL techniques, it has the limitation that generated synthetic tests may not fully reflect the execution semantics of real tests. FLITSR [17] addresses multi-fault scenarios in which one dominant fault masks the detection of others, adopting a strategy of iteratively reducing the test suite to isolate each fault individually. However, it requires that failing tests be provided in advance, making it inapplicable in environments where tests are absent. SBEST [18] notes that, in practice, most bugs lack fault-triggering tests, and it utilizes stack traces included in bug reports as a proxy for failing tests. Nonetheless, it is applicable only to bug reports that contain stack traces, and accuracy may degrade when the stack trace does not directly point to the fault location.

### 2.2. Information Retrieval-Based Fault Localization

IRFL ranks faulty files by treating bug reports as queries and source files as documents and measuring textual similarity, with the characteristic of being applicable without separate test execution. Bug2Commit [19] validates IRFL at an industrial scale; it integrates diverse features such as stack traces, exception messages, and performance regression metrics into a commit-level fault localization system based on a vector space model (VSM), which was deployed at Facebook. However, it relies on Facebook-specific infrastructure and large quantities of labeled historical data, limiting generalization to other environments. LLMiRO [20] addresses the core limitation of IRFL—inaccurate query construction—by leveraging GPT-4 to classify bug reports by type and applying type-appropriate query strategies and conversational query reformulation. However, dependence on the GPT-4 application programming interface (API) imposes constraints on cost and reproducibility. MACL-IRFL [21] treats as a problem the fact that existing IRFL considers only direct relationships between bug reports and source files; it models three relationships—between reports and code interactions, inter-report similarity, and inter-code co-citation—as a multi-view graph and suppresses irrelevant auxiliary information through contrastive learning. However, it requires historical bug-fix records for graph construction, and evaluation is limited to Java projects.

### 2.3. Learning-Based Fault Localization

LBFL aims to overcome the limitations of techniques that rely on statistical heuristics by learning complex relationships between software features and fault locations through neural network models. GRACE [22] notes that existing SBFL loses fine-grained coverage information and code structure information when compressing coverage into test counts or Boolean vectors; it constructs a single graph representing tests and program entities as nodes and coverage relationships and code structure as edges, and then ranks fault locations using a gated graph neural network (GGNN) [23]. However, GRACE still depends on coverage collection through test execution, and evaluation is limited to Java programs. TRANSFER [24] collects large-scale open-source bug data, trains a bidirectional long short-term memory (LSTM) [25]-based classifier to learn semantic features of faulty statements, and combines them with spectrum-based and mutation-based features. This approach supports both fault localization and automated program repair simultaneously, but it is restricted to 11 predefined bug types and incurs high training data construction costs. DepGraph [26] notes that GRACE focuses only on intra-procedural information and fails to leverage inter-procedural dependencies such as function call relationships; it is a graph neural network (GNN) model based on enhanced code representation that integrates an inter-procedural call graph into the code representation. However, DepGraph also presupposes test coverage collection and incurs additional analysis cost for inter-procedural graph construction.

### 2.4. LLM-Based Fault Localization

Agent-based approaches that leverage LLMs’ code understanding capability have emerged as a new paradigm for FL. AutoFL [9] enables autonomous repository exploration through function calling to overcome LLM context window limitations, and it generates a natural language explanation of the root cause along with the fault location. While it can be applied to both Java and Python with only a single failing test, the context window constraint remains a limitation for large codebase exploration, and the accuracy of generated explanations varies. LLMAO [27] fine-tunes bidirectional adapters on top of a pretrained LLM to enable line-level FL without test cases. However, the fine-tuning cost is high, and generalizability beyond the evaluated language and fault type scope has not been verified. AgentFL decomposes the FL process into three stages—fault understanding, codebase navigation, and fault confirmation—each executed by a specialized LLM agent, in order to handle project-level context that a single LLM cannot process. While it can handle project-scale context that is difficult for a single LLM, it has the limitations of error propagation risk across stages and LLM API dependency. Zhou et al. [2] empirically demonstrate that existing LLM agent-based FL techniques exhibit significantly degraded performance in the large-scale, limited-observability environment of the Linux kernel compared to general software, and they propose the LINUXFL+ framework incorporating directory-aware expansion and potential cause expansion strategies. However, evaluation is limited to the Linux kernel, and applicability to other large-scale systems requires additional verification.

ReAct-style agents interleave reasoning steps with external actions, allowing an LLM to form an intermediate hypothesis, request missing evidence through tools, and then refine the hypothesis based on the returned observation. This paradigm is suitable for kernel fault localization because kernel crash analysis naturally alternates between reasoning about a possible fault scenario and retrieving additional evidence from source code, call graphs, data structures, macros, and crash artifacts. Recent multi-agent systems such as MetaGPT [28] and AutoSizer [29] also show that specialized agents and tool-augmented iteration can improve complex engineering tasks. However, these systems are designed for general software development or design-space exploration, whereas AutoKFL targets Linux kernel crash localization. AutoKFL differs by grounding each ReAct loop in kernel-specific crash artifacts and by restricting agent actions to fault localization tasks such as crash observation, selective code collection, bug-pattern analysis, and evidence synthesis.

## 3. Proposal

**Figure 1:**
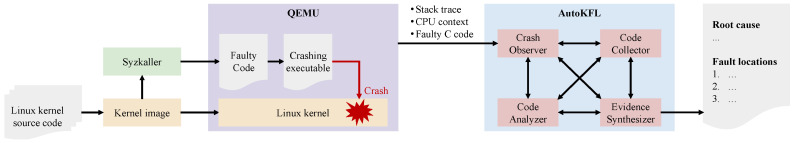
Overview of AutoKFL’s workflow.

Figure 1 illustrates the overall workflow of AutoKFL, a multi-agent fault localization system that automatically identifies the root cause and fault locations of Linux kernel crashes. AutoKFL takes as input three artifacts collected from a kernel crash event: a stack trace capturing the execution context at the time of the crash, a CPU context containing register states, and the C code that triggered the crash. These artifacts are obtained through dynamic crash reproduction under a controlled QEMU environment. AutoKFL does not use dynamic reproduction to perform runtime instrumentation or coverage-based localization; instead, it uses the reproduced artifacts as grounded evidence for subsequent static code retrieval and LLM-based reasoning. Thus, the contribution of dynamic reproduction is to stabilize and enrich the initial evidence provided to the agents, rather than to replace the agent-based localization process. Based on these inputs, four LLM-based agents participate in the analysis sequentially: the crash observer structures crash information, the code collector selectively gathers relevant kernel source code, the code analyzer detects bug patterns from the collected code and forms hypotheses about suspicious locations, and the evidence synthesizer integrates the outputs of all agents to produce the final result. AutoKFL’s output consists of a ranked list of fault locations expressed as file, function, and line number with a confidence score, along with a natural language root-cause analysis describing how the bug occurred.

The four agents of AutoKFL are derived by emulating the cognitive steps that human experts perform when analyzing kernel faults: observation, code collection, code analysis, and evidence synthesis. However, human experts do not always execute these steps in the same fixed order; they navigate directly to whichever step is needed based on analysis progress. A workflow that repeats a series of tasks in a fixed order is therefore insufficient for complex kernel code analysis [30,31,32,33]. A fixed workflow forces unnecessary steps to execute during analysis, wasting tokens and failing to respond flexibly to changes in analysis state. Based on this observation, AutoKFL adopts a flexible routing structure in which routing is determined by structured agent outputs rather than by a learned routing classifier. Each agent explicitly selects the next stage from a predefined set of valid destinations and provides a request message explaining what evidence is still needed. For example, the code analyzer routes to the code collector only when the collected code is insufficient to identify or rule out a bug location; otherwise, it routes to the evidence synthesizer.  The evidence synthesizer terminates the workflow when the accumulated evidence is sufficient for a ranked conclusion, or routes back to the code analyzer when the hypotheses are weak, incomplete, or contradictory.

### 3.1. Crash Observation

The crash observer is the first agent of AutoKFL, responsible for structuring crash information that serves as the foundation for all subsequent analysis. It receives as input a stack trace, CPU context, and the C code that triggered the crash, and it outputs structured information including the crash location (file, function, and line number), a list of suspicious functions, and related variable information. The core principle adhered to by the crash observer is the strict separation of observation and inference: this agent organizes collected information as-is and does not make any judgment about the root cause of the bug. This principle ensures that subsequent agents can conduct independent analysis based on unbiased information, ultimately improving the reliability of the overall analysis.

Structuring the stack trace in kernel crash analysis involves two difficulties: First, kernel call stacks contain frames unrelated to the actual fault—such as interrupt handlers, scheduler, and lock-related frames—so passing the raw stack trace to the LLM risks distracting it with irrelevant functions. Second, including the full source file at the crash point in the LLM’s context may exceed token limits due to the large size of kernel source files, or cause irrelevant code to degrade the LLM’s focus. These two problems mean that reliable crash information structuring is impossible with an approach that simply passes the raw stack trace and source files to the LLM.

**Figure 2:**
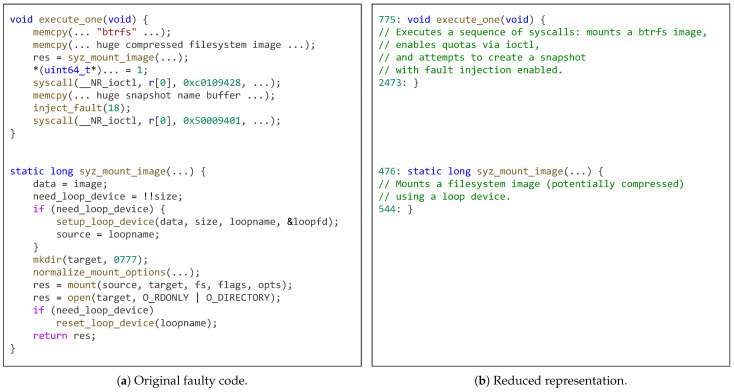
Example of code reduction.

To address these problems, the crash observer adopts two strategies: First, it uses an address-to-source mapping tool to convert the memory addresses contained in each frame of the call stack to exact source file paths, function names, and line numbers. This identifies frames directly related to the fault and excludes irrelevant kernel-internal frames from the analysis scope. Second, instead of including the raw source code at the crash point, it introduces a reduced faulty code representation. The faulty code generated by Syzkaller can easily exceed thousands of lines; providing such lengthy source code directly to an agent is likely to consume excessive tokens or be rejected due to context overflow. Reduced faulty code is a compressed summary that preserves function-level structure while omitting code blocks irrelevant to analysis. Figure 2 shows a comparison example of original source code and reduced faulty code. Through these two strategies, the crash observer provides subsequent agents with crash information that is both token-efficient and sufficient for analysis.

Through these two strategies, the crash observer provides subsequent agents with crash information that is both token-efficient and sufficient for initial analysis. Figure 2 shows one example of this reduction process: the representation keeps the function-level structure, original line ranges, and high-level crash-relevant operations, while omitting large code blocks that are unlikely to help the initial reasoning step. The exact contents retained in the reduced representation depend on the crash-triggering C code and the crash context; therefore, the reduction is not intended to be a complete semantic substitute for the original crash-triggering C code. If low-level argument values, initialization code, or auxiliary setup logic become relevant during later analysis, subsequent agents can request additional evidence through the code collector instead of relying only on the reduced faulty code.

### 3.2. Code Collection

The code collector is responsible for selectively gathering kernel source code needed for the code analyzer’s analysis, based on crash location and suspicious function information output by the crash observer. The collection targets consist of the source code of suspicious functions, data structure and macro definitions associated with those functions, inter-function call relationships, and data dependencies of key variables. Since the code analyzer requires sufficient collection of fault-related code to form accurate bug hypotheses, the quality of the code collector’s collection directly affects the accuracy of the overall analysis. The code collector must therefore maintain a balance between excluding code unnecessary for analysis and collecting code necessary for fault analysis without omission.

The Linux kernel is a vast codebase of tens of millions of lines, making it practically impossible to find crash-related code through exhaustive search. Collecting source code at the file level would include large amounts of irrelevant code, wasting tokens and reducing the LLM’s focus. Furthermore, which code is needed for fault analysis is difficult to determine before analysis begins; if the initial collection scope is insufficient, the code analyzer may fail to complete its analysis. This implies that a mechanism for dynamically expanding the collection scope is necessary.

**Figure 3:**
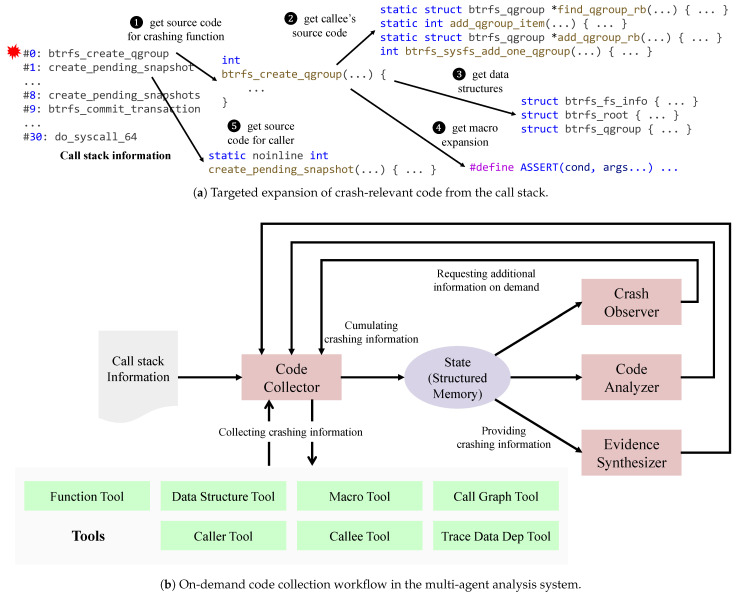
Code collection workflow of AutoKFL.

Figure 3a shows the targeted collection process of the code collector. Starting from the function at the crash location, it progressively expands the collection scope along call relationships in the callee and caller directions, and it additionally collects data structure and macro definitions referenced by each function. In this figure, numbering is shown in order from faulty function source code collection to caller source code collection for explanatory convenience, but the order is not mandatory since the code collector itself decides which task to perform next. Figure 3b shows the flow when additional collection is requested by the crash observer, code analyzer, or evidence synthesizer. In this case, the code collector expands the collection scope to the symbols specified in the request, stores results in a state repository implemented as structured memory [34] for subsequent reuse, and returns them to the requesting agent. By combining symbol-level selective collection with a dynamic expansion mechanism, the code collector ensures collection flexibility while securing the code needed for analysis with minimal token usage.

As shown in Figure 3, the code collector consists of seven exploration tools that operate at different granularities. The Function Tool retrieves the body and line range of crash-related functions. The Data Structure Tool and Macro Tool collect type and macro definitions required to interpret the retrieved code. The Call Graph Tool constructs caller–callee relationships around suspicious functions, while the Caller Tool and Callee Tool expand the search scope upward and downward along the call chain. The Trace Data Dependency Tool tracks variable definitions, uses, field accesses, pointer dereferences, and argument-passing sites within a function. Together, these tools allow the collector to expand evidence selectively at the symbol, call-relation, and variable-dependency levels rather than loading entire source files.

### 3.3. Code Analysis

The code analyzer is responsible for analyzing the kernel source code collected by the code collector to identify the root cause of faults. For each suspicious location, it formulates a bug scenario that concretely describes under what conditions and what kind of bug occurs, and computes a confidence score for each hypothesis. LLM code understanding and reasoning capabilities are utilized to trace the logical flow of source code and identify potential fault locations. The output of the code analyzer serves as the primary basis for the evidence synthesizer to compute the final fault location ranking.

Kernel bugs manifest in diverse patterns—such as null pointer dereference, use-after-free, locking violation, and race condition—each requiring different analysis perspectives. Failure to sufficiently examine all patterns in a single analysis pass may result in false positives or missed actual fault locations. Additionally, situations arise during analysis where the root cause cannot be clearly identified from only the initially collected code; this is particularly pronounced when faults span multiple functions or are located upstream in a data dependency chain. Reaching conclusions under insufficient evidence generates bug hypotheses with low confidence, which can distort subsequent judgments by the evidence synthesizer.

**Figure 4:**
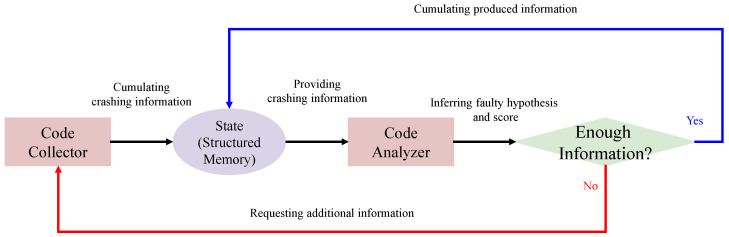
Iterative code analysis process.

Figure 4 shows the analysis loop of the code analyzer. The code analyzer formulates a bug hypothesis based on currently collected code, and then self-evaluates whether evidence is sufficient to support the hypothesis. If the evidence is judged sufficient, it finalizes and outputs the bug hypothesis and confidence score; if insufficient, it requests additional collection from the code collector and resumes analysis based on the returned code. Through this iterative refinement structure, the code analyzer autonomously resolves evidence insufficiency problems that may arise in a single analysis pass. The analysis results are cumulative, and when multiple analyses are performed, all results are forwarded to the evidence synthesizer for use in the final judgment.

### 3.4. Evidence Synthesis

The evidence synthesizer is the final agent of AutoKFL, responsible for integrating the outputs of the crash observer, code collector, and code analyzer to produce the final fault location ranking and root-cause explanation. The fault location ranking consists of file, function, and line number along with a confidence score, and the root-cause explanation describes in natural language understandable to developers how the bug occurred and which path led to the crash. The evidence synthesizer does not simply aggregate existing analysis results; it evaluates consistency and contradictions among results before rendering a final judgment. Since this agent’s output constitutes the final result of AutoKFL as a whole, rigorously evaluating the sufficiency of evidence and the reliability of conclusions is paramount.

When multiple code analysis results have accumulated, each may point to different suspicious locations or present contradictory hypotheses, necessitating a criterion for integration. An ensemble approach of repeated execution with the same input—as employed by AutoFL—is not practically applicable given the high cost structure of kernel crash analysis. Therefore, a constraint exists that a reliable ranking must be produced based only on analysis results accumulated within a single execution. This implies the need for an integration criterion that considers both the reliability of individual analysis results and the consistency among results.

The confidence value is a model-generated ranking score in the range of 0.0 to 1.0, produced by the evidence synthesizer for each ranked fault location. It is not a calibrated probability; rather, it represents the relative strength of support from the accumulated analysis context. The evidence synthesizer assigns this value by synthesizing the crash observation, collected code, and code-analysis results, and it ranks locations according to the apparent consistency and strength of the available evidence, such as crash-stack relevance, source-code support, and the causal explanation produced by the code analyzer. Therefore, a higher confidence value indicates that the location is better supported as a triage candidate, but it should not be interpreted as a statistically calibrated likelihood of being faulty. The root-cause explanation is generated by describing under what conditions and through what path the bug led to the crash, centered on the highest-ranked fault locations. When the currently available evidence is judged insufficient for a reliable conclusion, or when contradictions among analysis results remain unresolved, the evidence synthesizer requests re-analysis from the code analyzer or additional collection from the code collector to reinforce the analysis. Through this structure, the evidence synthesizer produces a grounded fault location ranking and root-cause explanation even under the constraint of a single execution.

## 4. Evaluation

To objectively assess whether the design and implementation of AutoKFL practically contribute to FL of the Linux kernel, this section describes the experiments organized around the dataset description and three research questions (RQs):

- RQ1: How much does each agent contribute to fault analysis of the Linux kernel?
- RQ2: How does analysis accuracy vary by crash type and kernel subsystem?
- RQ3: How much more accurate are AutoKFL’s analysis results compared to single-LLM-call approaches?

### 4.1. Dataset Description

For this experiment, 208 Linux kernel crashes reported on Syzbot [35] for which fixes have already been committed were selected as the dataset. Syzbot is an infrastructure that continuously runs the Syzkaller fuzzer to automatically detect and publicly report Linux kernel crashes, providing reproducible crashes together with their fixing commits. Two reasons motivated the selection of fixed crashes as targets: First, since the actual fault location is clearly confirmed through the committed patch, AutoKFL’s analysis results can be objectively verified. Second, Syzbot systematically reports crashes across Linux subsystems and crash types, facilitating the construction of a diverse experimental dataset.

We selected crashes with committed fixes not because they are necessarily easier but because they provide the only reliable basis for objective file- and function-level evaluation. In kernel fault localization, unresolved reports often lack a verified root cause, making it impossible to determine whether a predicted location is correct without subjective judgment. Fixing commits provide independently reviewed developer patches and, therefore, allow us to construct reproducible ground truth from real-world debugging outcomes. Although this selection does not cover unresolved crashes, it is a standard and necessary evaluation setting for measuring localization accuracy against verifiable fault locations.

Table A1 in Appendix A summarizes the 208 Linux kernel crashes used in this study, constructed to ensure diversity in terms of crash type and subsystem. The dataset covers 13 crash types, including general protection faults (GPF), kernel panic (PANIC), information leak (INFOLEAK), out-of-bounds access (OOB), deadlock, use-after-free (UAF), uninitialized value use (UNINIT), data race (DATARACE), memory leak (MEMLEAK), warning, null pointer dereference (NULL DEREF), hang (HANG), and general bug reports (BUG), with 16 cases for each type. By subsystem, networking accounts for the largest proportion, at 63 cases (30.3%), followed by filesystems at 54 cases (26.0%), memory management and drivers at 26 cases each (12.5%), others at 20 cases (9.6%), and block input and output (I/O) at 19 cases (9.1%). This dataset composition, covering diverse crash types and subsystems, is well suited for evaluating whether AutoKFL provides general fault localization capability across different bug characteristics and kernel components. The 208-crash dataset provides a substantially broader empirical basis for evaluation by covering multiple crash types and kernel subsystems. This dataset size reduces the risk that the reported performance reflects a small or narrowly selected set of crashes, and it allows the evaluation to characterize AutoKFL’s behavior across diverse Linux kernel fault scenarios.

### 4.2. Experimental Settings

All experiments were conducted on a single machine with an Intel Core i9-11900 at 2.50 GHz, 32 GB random access memory (RAM), and Ubuntu 24.04 long-term support (LTS) 64-bit. Gemini-3-flash, a model selected for its favorable cost–performance trade-off at the time of the experiments, was used as the base LLM of AutoKFL. Although higher-performing models such as GPT-5 and Claude Opus exist, this study aims to automate FL at a practical cost level, making it important to select a model with a balanced price-to-performance ratio. Gemini-3-flash was selected because it belonged to a lower-priced tier per million tokens at the time of the experiments, while still providing sufficient code reasoning capability for kernel crash analysis.

To improve reproducibility, all experiments used the same prompts, tool interfaces, routing schema, and evaluation procedure across all crash cases. The model name and experimental workflow were fixed during the evaluation period. Since commercial LLMs may change over time, we include the full agent prompts in Appendix B, Appendix C, Appendix D and Appendix E and report the evaluated model names explicitly, so that future work can reproduce the workflow structure even if exact model outputs vary.

The correctness of AutoKFL’s predictions was determined by comparison against the commit patch that fixes the corresponding crash. At the file level, the criterion was whether the file identified by AutoKFL as a fault location matches the file actually modified in the patch; at the function level, it was whether the identified function matches the function modified in the patch. However, since a single patch typically modifies code across multiple files and functions, it is difficult to accurately judge correctness by mechanically comparing against the list of modified locations. We therefore manually analyzed each patch to identify the modifications that resolve the core fault of the crash, and we set these as the ground truth. This process required domain knowledge to consider both the intent of the patch and the root cause of the crash, and it was performed under consistent criteria for all 208 crashes.

Ground-truth construction followed a two-stage curation and validation process. Two researchers first curated the dataset by inspecting the fixing commits, modified files and functions, commit messages, and crash context to identify the core fault-resolving locations. A third researcher, who was not involved in the initial curation, then independently validated the selected ground-truth files and functions. Cases with ambiguity, such as patches containing refactoring, validation logic, or cleanup code in addition to the root-cause fix, were resolved through discussion until consensus was reached. Because the process was based on curation followed by independent validation rather than fully independent parallel annotation of all cases, we did not compute a formal inter-annotator agreement score.

### 4.3. RQ1: Impact of Multi-Agent Architecture

To measure each agent’s contribution to overall FL performance, we conducted experiments constructing four ablation variants—each with one of the four agents removed—and comparing them against the full AutoKFL configuration. The evaluation metrics used were file-level and function-level Recall@N and mean reciprocal rank (MRR). Recall@N measures the proportion of cases in which the actual fault location is included in the top-N predictions. We measured performance varying N among 1, 3, and 5. MRR is the mean of the reciprocal of the rank at which the correct answer first appears, comprehensively reflecting ranking quality.

**Figure 5:**
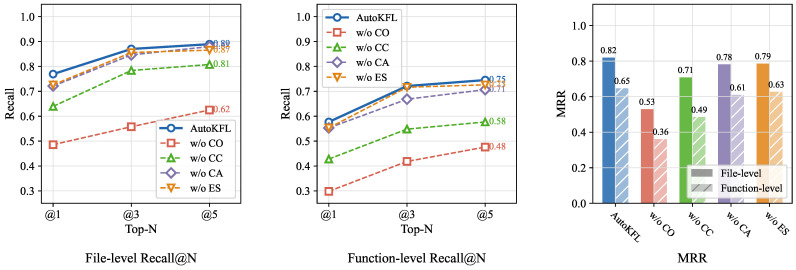
File-level Recall@N, Function-level Recall@N, and MRR for AutoKFL and ablation variants with each agent removed.

Figure 5 shows the Recall@N and MRR of each ablation variant, where w/o CO, w/o CC, w/o CA, and w/o ES indicate the removal of the crash observer, code collector, code analyzer, and evidence synthesizer, respectively. AutoKFL achieves the highest overall performance, with file-level Recall@5 of 0.89 and function-level Recall@5 of 0.75. The largest degradation occurs when the crash observer is removed: file-level Recall@5 drops to 0.62, function-level Recall@5 drops to 0.48, and file-level and function-level MRR decrease to 0.53 and 0.36, respectively. This result confirms that the crash observer plays a critical role in determining the initial analysis direction by structuring crash locations, suspicious functions, and crash-relevant context before subsequent code exploration.

Removing the code collector also causes a clear performance decrease, especially at the function level. The w/o CC variant achieves file-level Recall@5 of 0.81 and function-level Recall@5 of 0.58, with file-level and function-level MRR of 0.71 and 0.49, respectively. This indicates that crash-context information alone is insufficient for accurate localization, and that collected source-code evidence is necessary to connect the observed crash symptom to the actual faulty location. Removing the code analyzer or evidence synthesizer results in smaller performance drops than removing the crash observer or code collector. The w/o CA and w/o ES variants achieve file-level MRR values of 0.78 and 0.79 and function-level MRR values of 0.61 and 0.63, respectively. These results show that the complete AutoKFL pipeline provides the highest ranking quality among all evaluated configurations.

**Figure 6:**
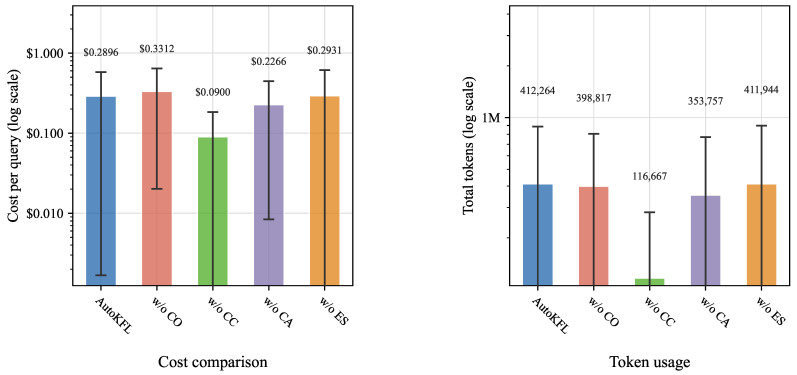
Cost per query and total token usage for AutoKFL and ablation variants.

Figure 6 shows the cost per query and total token usage of each ablation variant. AutoKFL costs $0.2896 per query on average and uses 412,264 tokens. The w/o CC variant shows the lowest cost and token usage, with $0.0900 per query and 116,667 tokens, because the source-code collection stage is removed. However, this cost reduction is accompanied by the performance degradation shown in Figure 5, demonstrating that code collection is not merely a cost factor but a necessary source of evidence for accurate localization. The w/o CA variant also reduces cost to $0.2266 and token usage to 353,757 tokens, but its ranking performance remains lower than that of AutoKFL. In contrast, removing the evidence synthesizer provides little cost benefit, with $0.2931 per query and 411,944 tokens, while still reducing MRR. The w/o CO variant even incurs the highest average cost, at $0.3312, showing that removing the initial observation stage does not necessarily improve cost efficiency because downstream agents must proceed with less structured crash information.

In summary, the ablation study confirms that all four agents make meaningful contributions to AutoKFL’s fault localization performance. The crash observer has the largest impact on accuracy because it defines the initial analysis direction, while the code collector provides the source-code evidence required for fine-grained localization. The code analyzer and evidence synthesizer further improve ranking quality by refining bug hypotheses and integrating accumulated evidence. Therefore, in response to RQ1, AutoKFL’s multi-agent architecture improves fault localization accuracy by assigning complementary roles to specialized agents, although this accuracy benefit comes with additional token and cost overheads compared with reduced ablation variants.

### 4.4. RQ2: Analysis by Bug Type and Kernel Subsystem

To analyze how AutoKFL’s performance varies by Linux kernel subsystem and crash type, we measured Recall@N and MRR broken down by subsystem and crash type. This enabled determination of whether AutoKFL provides general FL capability across different kernel components and crash characteristics, along with identification of the conditions under which performance limitations emerge.

Table 1 shows Recall@N and MRR by subsystem. AutoKFL achieves stable file-level performance across all subsystems, with file-level MRR ranging from 0.781 to 0.923. At the file level, Memory Mgmt shows the highest MRR of 0.923, followed by Driver with 0.869 and Others with 0.817. At the function level, Driver achieves the highest MRR of 0.849 and Recall@5 of 0.96, indicating that AutoKFL can localize faults precisely when the relevant driver-side execution context is well reflected in the collected crash and source-code evidence. In contrast, Filesystem and Others show lower function-level MRR values of 0.572 and 0.568, respectively. This indicates that fine-grained function-level localization remains more difficult when the fault is distributed across layered filesystem logic or heterogeneous kernel components.

The ablation results show that removing the crash observer causes the most consistent performance degradation across subsystems. For example, file-level MRR drops from 0.810 to 0.540 in Networking, from 0.781 to 0.454 in Filesystem, from 0.923 to 0.606 in Memory Mgmt, and from 0.817 to 0.475 in Others. A similar trend appears at the function level, where removing the crash observer reduces MRR by more than 0.23 in every subsystem. This confirms that the crash observer is critical for setting the initial analysis direction by structuring crash locations, suspicious functions, and crash-relevant context before source-code exploration begins. Removing the code collector also reduces performance, especially at the function level, showing that collected source-code evidence is necessary for fine-grained localization. In contrast, removing the code analyzer or evidence synthesizer generally results in smaller drops, although some subsystem-specific metrics slightly exceed the full configuration. This suggests that the contribution of each later-stage agent depends on the subsystem, while crash observation and code collection remain broadly important across kernel components.

**Figure 7:**
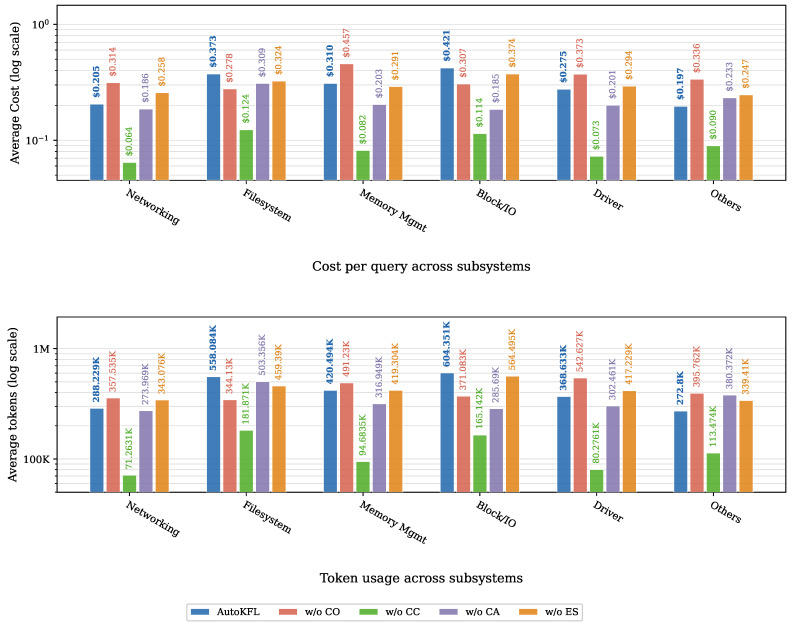
Average cost and token usage across subsystems for AutoKFL and ablation variants.

Figure 7 shows average cost per query and token usage by subsystem. For AutoKFL, Block I/O incurs the highest average cost and token usage, with $0.421 per query and 604,351 tokens, followed by Filesystem with $0.373 and 558,084 tokens. This indicates that Block I/O and Filesystem crashes require broader evidence collection or more iterative analysis than other subsystems. In contrast, Networking and Others require lower average costs, with $0.205 and $0.197 per query, respectively. The w/o CC variant consistently shows the lowest cost and token usage across all subsystems because the source-code collection stage is removed. However, as shown in Table 1, this reduction is accompanied by clear performance degradation, especially at the function level. Therefore, the cost analysis confirms that code collection is a major cost factor but also a necessary component for accurate localization.

Table 2 shows Recall@N and MRR by crash type. AutoKFL achieves high file-level performance for several crash types, including DATARACE with an MRR of 1.000, INFOLEAK and WARNING with 0.969, MEMLEAK with 0.938, and BUG with 0.891. At the function level, MEMLEAK achieves the highest MRR of 0.906, followed by WARNING with 0.877, INFOLEAK with 0.844, and DATARACE with 0.828. These results indicate that AutoKFL performs well when the crash report and collected code provide distinctive evidence for the faulty resource, access, or checked condition. However, PANIC shows the lowest performance, with file-level MRR of 0.419 and function-level MRR of 0.250. DEADLOCK also shows relatively low performance, with file-level MRR of 0.669 and function-level MRR of 0.512. These results suggest that crashes involving broad failure symptoms or synchronization-related conditions are more difficult to localize using the current static-artifact-centered workflow.

The crash-type ablation results again show the importance of the crash observer. Removing the crash observer sharply reduces performance for several crash types: MEMLEAK drops from 0.938 to 0.087 at the file level and from 0.906 to 0.062 at the function level, WARNING drops from 0.969 to 0.479 at the file level, and BUG drops from 0.891 to 0.433 at the file level. This indicates that initial crash structuring is especially important for crash types whose root cause must be inferred from report context and execution evidence. The code collector also contributes to fine-grained localization; for example, in Null pointer dereference, function-level MRR drops from 0.521 to 0.245 when the code collector is removed. On the other hand, the full configuration does not dominate every crash type and metric. For OOB, DEADLOCK, GPF, and PANIC, some ablation variants slightly outperform AutoKFL on specific file-level or function-level metrics. This shows that the benefit of each agent is not uniform across all crash types, and that different crash characteristics interact differently with the multi-agent workflow.

In summary, AutoKFL provides robust performance across diverse subsystems and crash types, but the results also reveal clear category-dependent variation. Subsystem-level results show that Driver and Memory Mgmt are handled effectively, while Filesystem and Others remain more challenging at the function level. Crash-type results show strong performance for DATARACE, INFOLEAK, WARNING, MEMLEAK, and BUG, but lower performance for PANIC and DEADLOCK. Therefore, in response to RQ2, AutoKFL’s performance varies meaningfully by subsystem and crash type, and the ablation results indicate that crash observation and code collection are the most broadly important components for maintaining localization accuracy across diverse kernel crashes.

### 4.5. RQ3: Comparison Across LLMs

To evaluate the benefit of AutoKFL’s tool-augmented multi-agent workflow over lightweight direct prompting, GPT-5.4-mini, Gemini-3-flash, and Claude-Haiku-4.5 were set as single-LLM-call baselines and provided the same crash-level input as AutoKFL. Since AutoKFL internally uses Gemini-3-flash as the agent LLM, single baseline LLMs were selected from state-of-the-art LLMs with a similar per-million-token price range to Gemini-3-flash, to enable a fair comparison. This comparison measures the performance difference between using an LLM in a single-call setting and composing the same type of model into a tool-augmented multi-agent workflow.

Table 3 shows the overall performance on the entire dataset. AutoKFL achieves the best performance across all file-level and function-level metrics. At the file level, AutoKFL achieves Recall@1 of 0.77, Recall@5 of 0.89, and MRR of 0.822. In contrast, the best single-LLM baseline, Gemini-3-flash, achieves file-level Recall@1 of 0.48, Recall@5 of 0.57, and MRR of 0.514. At the function level, AutoKFL achieves Recall@1 of 0.58, Recall@5 of 0.75, and MRR of 0.650, whereas Gemini-3-flash achieves Recall@1 of 0.28, Recall@5 of 0.38, and MRR of 0.320. These results show that direct single-call prompting is insufficient for Linux kernel fault localization, even when a capable LLM is used. The improvement of AutoKFL indicates that iterative code retrieval, role-separated reasoning, and evidence synthesis are necessary for accurately localizing kernel faults beyond the information directly visible in crash reports.

**Figure 8:**
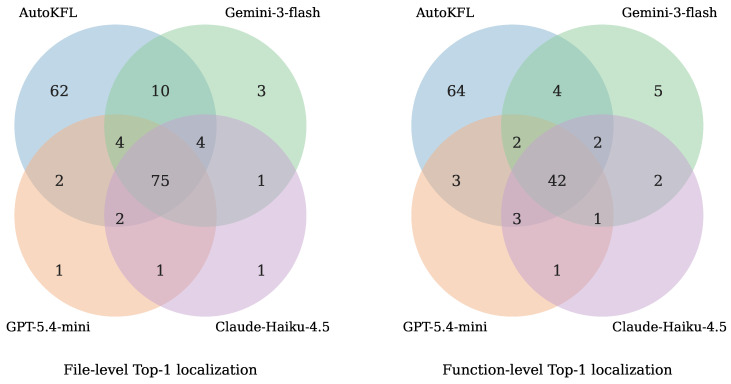
Overlap of correctly localized crashes at Top-1 among AutoKFL and single-LLM baselines at the file level and function level.

Figure 8 shows the overlap of crashes correctly localized by each method at the Top-1 criterion. At the file level, AutoKFL uniquely localizes 62 crashes that none of the single-LLM baselines correctly identify, while 75 crashes are correctly localized by all four methods. At the function level, AutoKFL uniquely localizes 64 crashes, while 42 crashes are correctly localized by all four methods. This result indicates that AutoKFL does not merely improve scores on cases already handled by single LLMs but also solves many additional cases that direct prompting fails to localize. The larger number of AutoKFL-only successes at the function level further suggests that the multi-agent workflow is particularly useful for fine-grained localization, where the faulty function may not be directly apparent from the crash symptom alone.

Table 4 shows the performance comparison by subsystem. AutoKFL outperforms all single-LLM baselines across all subsystems at both the file and function levels. At the file level, AutoKFL achieves MRR values of 0.809 in Networking, 0.781 in Filesystem, 0.923 in Memory Mgmt, 0.789 in Block I/O, 0.869 in Driver, and 0.817 in Others. The strongest single-LLM baseline varies by subsystem, but its MRR remains substantially lower than that of AutoKFL in every case. For example, in Memory Mgmt, AutoKFL achieves file-level MRR of 0.923, whereas Gemini-3-flash achieves 0.481 and Claude-Haiku-4.5 achieves 0.462. In Driver, AutoKFL achieves file-level MRR of 0.869 and function-level MRR of 0.849, while the strongest baseline, Gemini-3-flash, achieves 0.599 and 0.506, respectively. These results show that AutoKFL’s advantage is not limited to a specific subsystem but appears consistently across diverse kernel components.

The subsystem-level results also show that the performance gap becomes especially clear at the function level. In Filesystem, AutoKFL achieves function-level MRR of 0.572, whereas the strongest baseline achieves only 0.252. In Memory Mgmt, AutoKFL achieves function-level MRR of 0.663, whereas the strongest baseline reaches only 0.231. In Others, AutoKFL achieves function-level MRR of 0.568, whereas the strongest baseline reaches only 0.225. These gaps indicate that single-call LLMs can sometimes infer a plausible file from the crash context but often fail to identify the exact faulty function. In contrast, AutoKFL refines suspicious locations through crash observation, selective code collection, code analysis, and evidence synthesis, leading to better function-level ranking quality.

**Figure 9:**
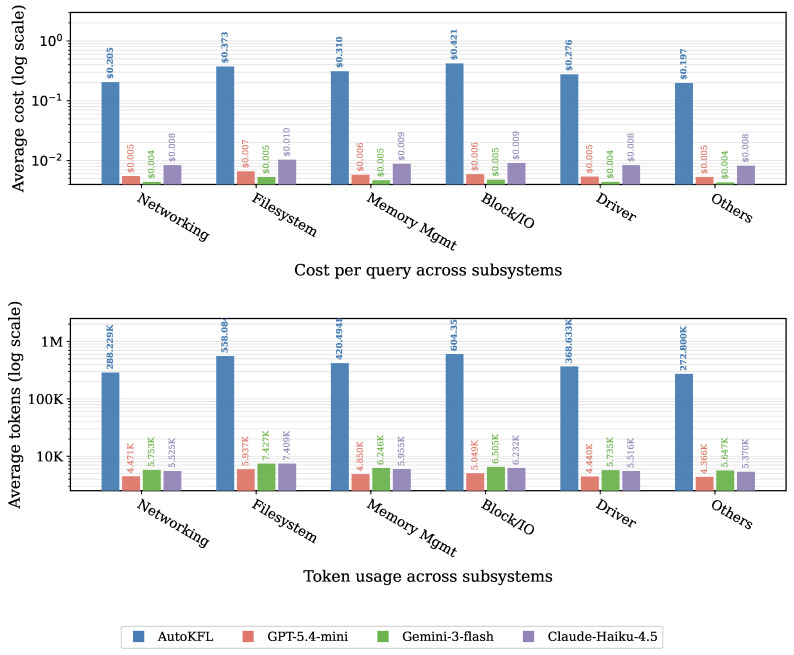
Average cost and token usage across subsystems for AutoKFL and single-LLM baselines.

Figure 9 shows the average cost per query and token usage by subsystem. AutoKFL incurs substantially higher cost and token usage than all single-LLM baselines because it performs iterative tool-augmented analysis rather than a single prompt completion. For AutoKFL, Block I/O incurs the highest average cost and token usage, with $0.421 per query and 604,351 tokens, followed by Filesystem with $0.373 and 558,084 tokens. Networking and Others incur lower costs, with $0.205 and $0.197 per query, respectively. This variation indicates that AutoKFL’s analysis cost depends on subsystem-specific difficulty, because more complex crashes require broader code collection and additional reasoning iterations. Single-LLM baselines remain much cheaper because they perform only one direct inference step, but this cost reduction comes with the substantial accuracy loss shown in Table 3 and Table 4.

Table 5 shows the performance comparison by crash type. AutoKFL achieves higher file-level MRR than all single-LLM baselines for every crash type. The performance advantage is especially large for crash types whose root causes are not directly exposed by the initial crash symptom. For example, in MEMLEAK, AutoKFL achieves file-level MRR of 0.938 and function-level MRR of 0.906, whereas the strongest single-LLM baseline reaches only 0.146 and 0.031, respectively. In INFOLEAK, AutoKFL achieves file-level MRR of 0.969 and function-level MRR of 0.844, whereas the strongest baseline reaches only 0.479 and 0.245, respectively. Similarly, in BUG, AutoKFL achieves file-level MRR of 0.891 and function-level MRR of 0.781, while the strongest baseline reaches only 0.510 and 0.396, respectively. These results indicate that single-call prompting often fails when fault localization requires reasoning from crash evidence to hidden resource, state, or validation logic.

AutoKFL also improves performance for difficult control-flow or synchronization-related crash types. For DEADLOCK, AutoKFL achieves file-level MRR of 0.669 and function-level MRR of 0.512, outperforming Gemini-3-flash, which achieves 0.578 and 0.359, respectively. For HANG, AutoKFL achieves file-level MRR of 0.786 and function-level MRR of 0.568, whereas the strongest single-LLM baseline reaches only 0.271 and 0.224, respectively. For PANIC, all methods show relatively low performance, and AutoKFL achieves file-level MRR of 0.419 and function-level MRR of 0.250. This suggests that PANIC remains challenging even for the full multi-agent workflow, because the reported failure symptom can be broad and may not directly reveal the root-cause location. Nevertheless, AutoKFL still provides the highest file-level ranking quality for PANIC and consistently improves over single-call baselines across most fine-grained metrics.

For crash types with clearer evidence, the gap between AutoKFL and single LLMs becomes smaller but remains consistent. For DATARACE, AutoKFL achieves perfect file-level MRR of 1.000 and function-level MRR of 0.828, while the strongest baseline achieves 0.927 and 0.658, respectively. For OOB, AutoKFL achieves file-level MRR of 0.781 and function-level MRR of 0.583, whereas the strongest baseline achieves 0.688 and 0.490, respectively. For GPF, AutoKFL achieves file-level MRR of 0.781 and function-level MRR of 0.677, whereas the strongest baseline achieves 0.688 and 0.325, respectively. These results show that AutoKFL remains beneficial even when single LLMs can infer reasonable candidates, because the multi-agent workflow improves ranking quality through additional source-code evidence and synthesis.

**Figure 10:**
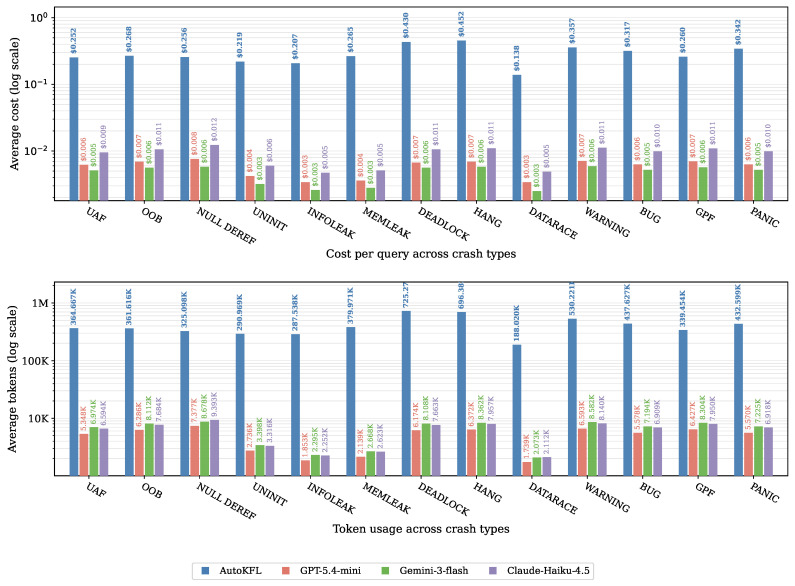
Average cost and token usage across crash types for AutoKFL and single-LLM baselines.

Figure 10 shows the average cost and token usage by crash type. AutoKFL incurs the highest cost for HANG and DEADLOCK, with $0.452 and $0.430 per query, respectively. These two crash types also require the largest token usage, with 696,382 tokens for HANG and 725,271 tokens for DEADLOCK. This suggests that synchronization-related or non-local failure symptoms require broader evidence collection and more iterative reasoning. In contrast, DATARACE shows the lowest AutoKFL cost, with $0.138 per query and 188,020 tokens. Single-LLM baselines remain consistently inexpensive across crash types, generally using only a few thousand tokens per query. However, their low cost reflects the absence of iterative code retrieval and evidence synthesis, which also explains their lower localization accuracy.

In summary, AutoKFL provides consistent performance improvements over single-LLM-call approaches across the full dataset, across subsystems, and across crash types. The improvement is especially large at the function level, where accurate localization requires more than directly interpreting the crash report. The overlap analysis further shows that AutoKFL correctly localizes many crashes that none of the single LLMs can localize at Top-1. However, these accuracy gains come with substantially higher inference cost and token usage, especially for difficult categories such as Block I/O, Filesystem, HANG, and DEADLOCK. Therefore, in response to RQ3, the results show that AutoKFL’s complete workflow is more effective than direct single-call prompting under the evaluated setting, especially for difficult crashes that require iterative code retrieval and evidence synthesis. This comparison should be interpreted not as isolating every individual design choice in AutoKFL but as demonstrating the practical benefit of the proposed workflow as a whole.

### 4.6. Case Study

To further interpret the quantitative results, we present representative case studies from AutoKFL’s analysis traces. These cases illustrate both how AutoKFL can connect crash symptoms to the actual fixing location and where the current workflow still fails to rank the root cause correctly.

#### 4.6.1. Correct Root-Cause Localization

**Figure 11:**
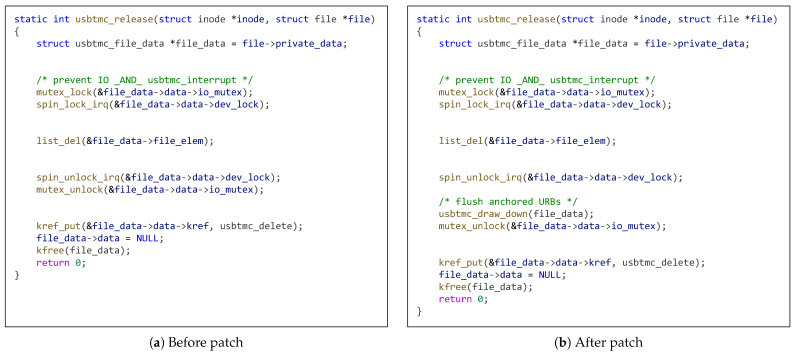
Patch excerpt from drivers/usb/class/usbtmc.c for report 9a3c54f52bd1edbd975f.

Figure 11 shows the fixing patch for report 9a3c54f52bd1edbd975f, a UAF crash reported by Syzbot in usb_anchor_suspend_wakeups. Although the crash is observed in the universal serial bus (USB) core, the patch modifies usbtmc_release in drivers/usb/class/usbtmc.c. Specifically, the fix inserts usbtmc_draw_down(file_data) before releasing the file-specific data structure. This patch indicates that the root cause is not the crash-surfacing access itself but the missing cleanup of anchored USB request blocks (URBs) before file_data is released.

AutoKFL localized this case by progressively connecting the crash stack with the driver-side resource lifetime. The crash observer first identified usb_anchor_suspend_wakeups, usb_unanchor_urb, __usb_hcd_giveback_urb, and usbtmc_release as suspicious functions from the crash report and call stack. The code collector then gathered the relevant USB core functions, the usbtmc_release implementation, and the usb_anchor and usbtmc_file_data data structures. Using this evidence, the code analyzer inferred that URBs anchored to fields inside file_data could remain in flight when usbtmc_release frees the structure.

The final synthesis ranked usbtmc_release as the most likely fault location, with usb_anchor_suspend_wakeups retained as the crash manifestation site. This ranking matches the fixing commit shown in Figure 11, where the added usbtmc_draw_down call flushes anchored URBs before the subsequent release path proceeds. This case illustrates that AutoKFL can distinguish between the function where a memory error is detected and the function responsible for the invalid object lifetime. Therefore, the result supports the usefulness of combining crash observation, selective code collection, and evidence synthesis for root-cause localization in kernel driver crashes.

#### 4.6.2. Mislocalization Case

**Figure 12:**
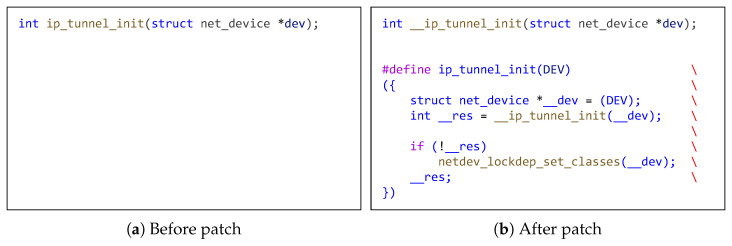
Patch excerpt from include/net/ip_tunnels.h for report 1240b33467289f5ab50b.

Figure 12 shows the fixing patch for report 1240b33467289f5ab50b, which was reported as a possible deadlock in sch_direct_xmit. The patch changes ip_tunnel_init() from a shared Internet Protocol version 4 (IPv4) tunnel initializer into a macro that invokes netdev_lockdep_set_classes() at each call site. The corresponding implementation in net/ipv4/ip_tunnel.c is renamed to __ip_tunnel_init(), and the direct lock-class assignment is removed from the shared initializer. This indicates that the actual fault is related to tunnel initialization-time lock-class assignment, rather than the packet transmission function where the warning is reported.

AutoKFL partially inferred this mechanism during the analysis. The crash observer identified sch_direct_xmit, ip_tunnel_xmit, and ipgre_xmit as suspicious functions from the lockdep warning and the call stack. The code collector then gathered the tunnel transmission path, the ip_tunnel_init macro, __ip_tunnel_init, and netdev_lockdep_set_classes. Based on this evidence, AutoKFL recognized that nested generic routing encapsulation (GRE) and encapsulated remote SPAN (ERSPAN) tunnels could share lock classes and trigger a lockdep warning.

However, the final ranking still placed sch_direct_xmit in net/sched/sch_generic.c as the top-ranked location. The actual patch shown in Figure 12 modifies include/net/ip_tunnels.h and net/ipv4/ip_tunnel.c, while sch_direct_xmit is only the site where lockdep reports the warning. Although include/net/ip_tunnels.h appeared as a lower-ranked candidate, the top-ranked file did not match the fixing location. Thus, this case is counted as a file-level Top-1 mislocalization.

This case illustrates a limitation of AutoKFL for bugs whose root cause is separated from the crash-reporting stack by initialization or configuration logic. The analysis captured the high-level lock-class issue but assigned excessive importance to the runtime warning site. In such cases, the stack trace provides strong evidence about where the symptom is detected, while the actual fix may reside in a shared initialization path that is not directly responsible for the final lock acquisition. Therefore, this failure suggests that AutoKFL should more explicitly distinguish between symptom locations and configuration sites when analyzing lockdep-related kernel reports.

## 5. Discussion

The experimental results allow several meaningful observations to be derived regarding AutoKFL’s design principles and limitations. This section discusses key findings confirmed through experiments and limitations of AutoKFL. Analysis of experimental results across the three research questions confirms that AutoKFL’s multi-agent design practically contributes to FL as follows:

- AutoKFL exhibits overall higher performance compared to single-LLM-call approaches, with the contribution of the crash observer most prominent. This suggests that the initial observation stage, which structures crash information and forwards it to subsequent agents, determines the direction of the entire analysis. Furthermore, the fact that cost also decreased most significantly when the code collector was removed, while performance degraded simultaneously, demonstrates that selective code collection is not merely a cost item but a core element of analysis quality.
- Across experiments, AutoKFL’s performance and cost varied by subsystem. Filesystem and Others showed relatively lower function-level MRR, while Block I/O and Filesystem required higher cost and token usage. This suggests that layered subsystem logic and heterogeneous kernel components make fine-grained localization more difficult and often require broader evidence collection.
- AutoKFL tends to exhibit higher performance at the file level than at the function level. This gap arises because the file containing the relevant fault can often be inferred from the crash stack and surrounding call path, whereas the exact faulty function may be separated from the crash-surfacing function by wrappers, helper routines, callbacks, or cross-file data dependencies. In kernel crashes, a patch may also modify multiple functions for validation, cleanup, or state restoration, making the boundary between the root-cause function and supporting fix locations less clear. Thus, AutoKFL’s function-level results should be interpreted as fine-grained triage candidates rather than as a complete replacement for developer inspection.

On the other hand, the experiments also revealed structural and practical limitations that AutoKFL currently possesses, which serve as important pointers for future improvement.

- AutoKFL incurs substantially higher inference cost than single-LLM baselines because it performs iterative code collection, re-analysis, and evidence synthesis. Therefore, AutoKFL is not intended to replace lightweight single-call triage for all crash reports. Rather, it is most practical for high-priority kernel crashes, repeatedly reproduced crashes, or cases where file-level triage by a single LLM is insufficient and developers need a ranked explanation grounded in crash artifacts and source-code evidence. This cost–performance trade-off suggests that AutoKFL should be deployed as an escalation-stage analyzer rather than as a first-pass filter for every incoming report.
- The dataset used in this study consists of 208 fixed Syzbot reports whose fault locations can be verified through fixing commits. This selection enables objective file-level and function-level evaluation, but the dataset still reflects the distribution of fixed Syzbot reports available under our collection criteria. Future work should evaluate AutoKFL on additional kernel bug sources and broader subsystem distributions to further validate its generalizability.
- AutoKFL performs analysis based solely on static artifacts without runtime instrumentation. Therefore, accurate localization may be difficult for crashes in which timing dependency at execution time is the core cause of the fault, such as race conditions. In such cases, supplementing agent inputs with dynamic analysis results may be considered; however, dynamic instrumentation in the kernel environment entails separate technical challenges.
- AutoKFL currently assumes the availability of Syzkaller/Syzbot-style crash artifacts, including a reproducing program, stack trace, and crash-time context. This assumption is a limitation of the current study, because AutoKFL may not be directly applicable to kernel bugs with incomplete reports, unreliable reproducers, or environment-specific failures that cannot be reconstructed in QEMU. However, this limitation follows from the intended scope of AutoKFL: this work targets reproducible Linux kernel crashes for which sufficient crash evidence is available, and it focuses on fault localization rather than crash reproduction or report completion. We therefore leave the extension of AutoKFL to non-reproducible or under-specified kernel bugs as future work.
- Although our ablation study evaluates the contribution of each agent by removing one component at a time, it does not exhaustively explore all possible workflow variants, such as fixed routing, alternative retrieval scopes, prompt variants, or different tool combinations. We focus on component-level ablation because the goal of this study is to validate whether the proposed agent roles contribute to kernel fault localization. A more exhaustive design-space analysis of routing and retrieval policies is left for future work.

## 6. Conclusions

This paper proposes AutoKFL, an automated fault localization system for Linux kernel crashes. AutoKFL employs four LLM-based agents—crash observer, code collector, code analyzer, and evidence synthesizer—designed to emulate the analysis process of human experts. These agents collaborate to perform crash observation, code collection, code analysis, and evidence synthesis, with each agent operating in a ReAct manner and supporting iterative exploration through conditional routing that allows returning to a prior stage when necessary.

Experimental results on 208 fixed Syzbot reports show that AutoKFL provides consistent performance improvements over single-LLM-call approaches at both the file and function levels. Ablation experiments confirm that each agent contributes to localization performance and show that the crash observer and code collector play especially important roles in guiding the analysis direction and constructing source-code evidence. The results also show that AutoKFL is more effective for file-level localization than function-level localization, indicating that fine-grained faulty-function identification remains challenging in Linux kernel crashes.

Despite these advantages, AutoKFL still has practical and structural limitations. Its iterative multi-agent workflow incurs substantially higher inference cost than single-LLM baselines, making it more suitable as an escalation-stage analyzer than as a first-pass filter for every crash report. In addition, AutoKFL relies on static crash artifacts and assumes the availability of Syzkaller/Syzbot-style reproducing programs, stack traces, and crash-time context. Future work will investigate adaptive strategies that adjust analysis depth according to crash difficulty, incorporate lightweight dynamic evidence for timing-dependent bugs, and evaluate AutoKFL on broader kernel bug sources and subsystem distributions.

## Figures and Tables

**Table 1:** File-level and function-level Recall@N and MRR across subsystems for AutoKFL and ablation variants.

Subsystem	Variant	File-Level				Function-Level			
		R@1	R@3	R@5	MRR	R@1	R@3	R@5	MRR
	AutoKFL	0.75	0.87	0.87	0.810	0.57	0.71	0.73	0.647
	w/o CO	0.49	0.57	0.63	0.540	0.25	0.43	0.46	0.340
Networking	w/o CC	0.71	0.83	0.84	0.768	0.57	0.67	0.68	0.617
	w/o CA	0.71	0.84	0.87	0.782	0.56	0.68	0.71	0.627
	w/o ES	0.75	0.86	0.86	0.796	0.62	0.73	0.73	0.675
	AutoKFL	0.72	0.81	0.87	0.781	0.48	0.67	0.69	0.572
	w/o CO	0.39	0.48	0.59	0.454	0.26	0.33	0.43	0.312
Filesystem	w/o CC	0.50	0.69	0.74	0.594	0.35	0.46	0.52	0.414
	w/o CA	0.65	0.81	0.83	0.723	0.46	0.59	0.65	0.528
	w/o ES	0.65	0.78	0.80	0.714	0.43	0.63	0.65	0.522
	AutoKFL	0.88	0.96	0.96	0.923	0.62	0.69	0.73	0.663
	w/o CO	0.58	0.62	0.65	0.606	0.27	0.35	0.46	0.328
Memory Mgmt	w/o CC	0.69	0.85	0.88	0.772	0.31	0.50	0.54	0.399
	w/o CA	0.81	0.88	0.92	0.854	0.58	0.62	0.69	0.613
	w/o ES	0.81	0.96	0.96	0.878	0.50	0.69	0.69	0.577
	AutoKFL	0.79	0.79	0.79	0.789	0.63	0.74	0.74	0.675
	w/o CO	0.53	0.63	0.63	0.570	0.37	0.53	0.53	0.439
Block I/O	w/o CC	0.63	0.74	0.74	0.684	0.47	0.47	0.47	0.474
	w/o CA	0.79	0.79	0.89	0.813	0.63	0.68	0.74	0.668
	w/o ES	0.74	0.79	0.79	0.763	0.63	0.74	0.79	0.695
	AutoKFL	0.81	0.92	0.96	0.869	0.77	0.92	0.96	0.849
	w/o CO	0.58	0.62	0.73	0.621	0.54	0.62	0.69	0.592
Driver	w/o CC	0.73	0.85	0.85	0.788	0.50	0.65	0.69	0.587
	w/o CA	0.81	0.96	1.00	0.873	0.77	0.92	0.92	0.833
	w/o ES	0.77	0.92	0.96	0.849	0.73	0.92	0.92	0.821
	AutoKFL	0.75	0.90	0.90	0.817	0.50	0.65	0.70	0.568
	w/o CO	0.45	0.50	0.50	0.475	0.20	0.35	0.35	0.258
Others	w/o CC	0.60	0.80	0.80	0.700	0.20	0.40	0.40	0.283
	w/o CA	0.65	0.80	0.80	0.725	0.40	0.55	0.55	0.458
	w/o ES	0.70	0.90	0.90	0.800	0.45	0.65	0.65	0.542

**Table 2:** File-level and function-level Recall@N and MRR across crash types for AutoKFL and ablation variants.

Crash Type	Variant	File-Level				Function-Level			
		R@1	R@3	R@5	MRR	R@1	R@3	R@5	MRR
UAF	AutoKFL	0.81	0.88	0.88	0.844	0.44	0.50	0.56	0.474
	w/o CO	0.56	0.69	0.75	0.630	0.25	0.38	0.50	0.330
	w/o CC	0.56	0.81	0.81	0.677	0.25	0.44	0.50	0.346
	w/o CA	0.75	0.88	0.88	0.812	0.31	0.50	0.62	0.434
	w/o ES	0.69	0.81	0.81	0.750	0.38	0.56	0.56	0.458
OOB	AutoKFL	0.69	0.88	0.88	0.781	0.44	0.75	0.75	0.583
	w/o CO	0.81	0.88	0.88	0.844	0.44	0.69	0.75	0.557
	w/o CC	0.75	0.88	0.88	0.812	0.44	0.69	0.69	0.552
	w/o CA	0.69	0.88	0.88	0.781	0.50	0.56	0.69	0.562
	w/o ES	0.75	0.88	0.88	0.802	0.56	0.75	0.75	0.646
NULL DEREF	AutoKFL	0.69	0.88	0.88	0.771	0.44	0.62	0.62	0.521
	w/o CO	0.62	0.69	0.69	0.656	0.38	0.56	0.56	0.458
	w/o CC	0.50	0.69	0.69	0.594	0.19	0.31	0.38	0.245
	w/o CA	0.62	0.81	0.94	0.747	0.38	0.56	0.62	0.471
	w/o ES	0.69	0.81	0.81	0.750	0.56	0.62	0.62	0.594
UNINIT	AutoKFL	0.81	0.94	0.94	0.875	0.50	0.75	0.75	0.625
	w/o CO	0.56	0.56	0.69	0.591	0.31	0.44	0.50	0.380
	w/o CC	0.62	0.75	0.75	0.688	0.44	0.56	0.62	0.502
	w/o CA	0.75	0.81	0.88	0.786	0.56	0.62	0.69	0.609
	w/o ES	0.75	0.88	0.88	0.802	0.56	0.75	0.75	0.646
INFOLEAK	AutoKFL	0.94	1.00	1.00	0.969	0.81	0.88	0.88	0.844
	w/o CO	0.56	0.56	0.56	0.562	0.50	0.50	0.50	0.500
	w/o CC	0.56	0.75	0.75	0.656	0.38	0.44	0.44	0.406
	w/o CA	0.62	0.88	0.88	0.729	0.56	0.62	0.62	0.594
	w/o ES	0.75	0.88	0.94	0.828	0.62	0.75	0.75	0.688
MEMLEAK	AutoKFL	0.94	0.94	0.94	0.938	0.88	0.94	0.94	0.906
	w/o CO	0.06	0.06	0.19	0.087	0.06	0.06	0.06	0.062
	w/o CC	0.75	0.81	0.81	0.771	0.56	0.62	0.62	0.594
	w/o CA	0.94	0.94	1.00	0.950	0.81	0.88	0.88	0.844
	w/o ES	0.94	0.94	0.94	0.938	0.75	0.94	0.94	0.833
DEADLOCK	AutoKFL	0.56	0.75	0.81	0.669	0.38	0.62	0.69	0.512
	w/o CO	0.44	0.56	0.69	0.525	0.19	0.38	0.44	0.294
	w/o CC	0.56	0.62	0.69	0.609	0.38	0.44	0.50	0.422
	w/o CA	0.56	0.75	0.81	0.648	0.38	0.62	0.69	0.492
	w/o ES	0.62	0.88	0.88	0.740	0.44	0.69	0.69	0.552
HANG	AutoKFL	0.75	0.81	0.88	0.786	0.50	0.62	0.69	0.568
	w/o CO	0.31	0.38	0.38	0.344	0.25	0.31	0.31	0.281
	w/o CC	0.50	0.69	0.75	0.599	0.31	0.44	0.44	0.375
	w/o CA	0.69	0.81	0.88	0.752	0.44	0.62	0.62	0.500
	w/o ES	0.56	0.81	0.81	0.667	0.38	0.69	0.75	0.523
DATARACE	AutoKFL	1.00	1.00	1.00	1.000	0.81	0.81	0.88	0.828
	w/o CO	0.81	0.94	1.00	0.880	0.38	0.69	0.81	0.535
	w/o CC	0.88	1.00	1.00	0.927	0.62	0.81	0.88	0.721
	w/o CA	0.94	1.00	1.00	0.969	0.75	0.81	0.88	0.797
	w/o ES	0.88	1.00	1.00	0.938	0.62	0.88	0.88	0.750
WARNING	AutoKFL	0.94	1.00	1.00	0.969	0.81	0.94	1.00	0.877
	w/o CO	0.38	0.56	0.69	0.479	0.25	0.31	0.38	0.286
	w/o CC	0.75	0.94	0.94	0.844	0.62	0.81	0.81	0.708
	w/o CA	0.81	1.00	1.00	0.896	0.75	0.94	0.94	0.833
	w/o ES	1.00	1.00	1.00	1.000	0.81	0.88	0.88	0.833
BUG	AutoKFL	0.81	0.94	1.00	0.891	0.69	0.88	0.88	0.781
	w/o CO	0.38	0.44	0.62	0.433	0.31	0.38	0.50	0.369
	w/o CC	0.69	0.88	1.00	0.792	0.56	0.62	0.69	0.609
	w/o CA	0.75	0.88	0.88	0.802	0.75	0.75	0.75	0.750
	w/o ES	0.56	0.94	1.00	0.762	0.50	0.75	0.81	0.637
GPF	AutoKFL	0.69	0.88	0.88	0.781	0.56	0.81	0.81	0.677
	w/o CO	0.56	0.69	0.75	0.641	0.31	0.50	0.62	0.427
	w/o CC	0.81	0.88	0.88	0.833	0.62	0.69	0.69	0.656
	w/o CA	0.75	0.88	0.88	0.812	0.69	0.88	0.88	0.771
	w/o ES	0.88	0.94	0.94	0.906	0.75	0.81	0.81	0.781
PANIC	AutoKFL	0.38	0.44	0.50	0.419	0.25	0.25	0.25	0.250
	w/o CO	0.25	0.25	0.25	0.250	0.25	0.25	0.25	0.250
	w/o CC	0.38	0.50	0.56	0.450	0.19	0.25	0.25	0.208
	w/o CA	0.50	0.50	0.56	0.512	0.31	0.31	0.31	0.312
	w/o ES	0.38	0.38	0.38	0.375	0.25	0.25	0.25	0.250

**Table 3:** File-level and function-level Recall@N and MRR for AutoKFL and single-LLM baselines.

Method	File-Level				Function-Level			
	R@1	R@3	R@5	MRR	R@1	R@3	R@5	MRR
AutoKFL	0.77	0.87	0.89	0.822	0.58	0.72	0.75	0.650
GPT-5.4-mini	0.41	0.47	0.50	0.447	0.25	0.30	0.35	0.284
Gemini-3-flash	0.48	0.56	0.57	0.514	0.28	0.36	0.38	0.320
Claude-Haiku-4.5	0.41	0.48	0.50	0.447	0.25	0.35	0.37	0.295

**Table 4:** File-level and function-level Recall@N and MRR across subsystems for AutoKFL and single-LLM baselines.

Subsystem	Method	File-Level				Function-Level			
		R@1	R@3	R@5	MRR	R@1	R@3	R@5	MRR
Networking	AutoKFL	0.75	0.87	0.87	0.809	0.57	0.71	0.73	0.647
	GPT-5.4-mini	0.43	0.52	0.59	0.486	0.29	0.38	0.41	0.336
	Gemini-3-flash	0.52	0.65	0.65	0.577	0.33	0.41	0.43	0.371
	Claude-Haiku-4.5	0.44	0.54	0.59	0.499	0.25	0.41	0.43	0.332
Filesystem	AutoKFL	0.72	0.81	0.87	0.781	0.48	0.67	0.69	0.572
	GPT-5.4-mini	0.39	0.43	0.44	0.412	0.20	0.24	0.28	0.228
	Gemini-3-flash	0.41	0.50	0.52	0.446	0.19	0.28	0.31	0.238
	Claude-Haiku-4.5	0.37	0.44	0.48	0.410	0.20	0.30	0.33	0.252
Memory Mgmt	AutoKFL	0.88	0.96	0.96	0.923	0.62	0.69	0.73	0.663
	GPT-5.4-mini	0.35	0.42	0.50	0.404	0.12	0.15	0.23	0.152
	Gemini-3-flash	0.42	0.54	0.54	0.481	0.19	0.27	0.27	0.231
	Claude-Haiku-4.5	0.42	0.50	0.50	0.462	0.15	0.23	0.23	0.186
Block I/O	AutoKFL	0.79	0.79	0.79	0.789	0.63	0.74	0.74	0.675
	GPT-5.4-mini	0.42	0.47	0.47	0.439	0.37	0.42	0.42	0.386
	Gemini-3-flash	0.47	0.53	0.53	0.500	0.32	0.37	0.42	0.353
	Claude-Haiku-4.5	0.42	0.47	0.47	0.447	0.32	0.42	0.42	0.360
Driver	AutoKFL	0.81	0.92	0.96	0.869	0.77	0.92	0.96	0.849
	GPT-5.4-mini	0.46	0.50	0.50	0.474	0.38	0.38	0.42	0.394
	Gemini-3-flash	0.58	0.62	0.65	0.599	0.46	0.54	0.62	0.506
	Claude-Haiku-4.5	0.35	0.42	0.42	0.378	0.42	0.46	0.46	0.436
Others	AutoKFL	0.75	0.90	0.90	0.817	0.50	0.65	0.70	0.568
	GPT-5.4-mini	0.45	0.45	0.45	0.450	0.15	0.20	0.30	0.200
	Gemini-3-flash	0.45	0.45	0.45	0.450	0.20	0.25	0.25	0.225
	Claude-Haiku-4.5	0.45	0.45	0.45	0.450	0.15	0.20	0.30	0.189

**Table 5:** File-level and function-level Recall@N and MRR across crash types for AutoKFL and single-LLM baselines.

Crash Type	Method	File-Level				Function-Level			
		R@1	R@3	R@5	MRR	R@1	R@3	R@5	MRR
UAF	AutoKFL	0.81	0.88	0.88	0.844	0.44	0.50	0.56	0.474
	GPT-5.4-mini	0.56	0.62	0.62	0.594	0.25	0.31	0.31	0.281
	Gemini-3-flash	0.56	0.62	0.62	0.594	0.25	0.31	0.31	0.281
	Claude-Haiku-4.5	0.44	0.62	0.62	0.531	0.19	0.31	0.31	0.240
OOB	AutoKFL	0.69	0.88	0.88	0.781	0.44	0.75	0.75	0.583
	GPT-5.4-mini	0.50	0.62	0.75	0.594	0.31	0.44	0.56	0.396
	Gemini-3-flash	0.56	0.81	0.81	0.677	0.38	0.62	0.62	0.490
	Claude-Haiku-4.5	0.62	0.69	0.81	0.688	0.31	0.56	0.62	0.443
NULL DEREF	AutoKFL	0.69	0.88	0.88	0.771	0.44	0.62	0.62	0.521
	GPT-5.4-mini	0.44	0.50	0.50	0.458	0.19	0.19	0.25	0.203
	Gemini-3-flash	0.50	0.50	0.50	0.500	0.25	0.25	0.31	0.266
	Claude-Haiku-4.5	0.50	0.50	0.50	0.500	0.25	0.25	0.31	0.266
UNINIT	AutoKFL	0.81	0.94	0.94	0.875	0.50	0.75	0.75	0.625
	GPT-5.4-mini	0.50	0.56	0.56	0.531	0.31	0.31	0.31	0.312
	Gemini-3-flash	0.50	0.62	0.62	0.552	0.25	0.38	0.38	0.312
	Claude-Haiku-4.5	0.50	0.56	0.62	0.547	0.31	0.38	0.38	0.333
INFOLEAK	AutoKFL	0.94	1.00	1.00	0.969	0.81	0.88	0.88	0.844
	GPT-5.4-mini	0.25	0.25	0.31	0.263	0.19	0.19	0.19	0.188
	Gemini-3-flash	0.44	0.56	0.56	0.479	0.19	0.31	0.38	0.245
	Claude-Haiku-4.5	0.12	0.25	0.25	0.177	0.19	0.19	0.19	0.188
MEMLEAK	AutoKFL	0.94	0.94	0.94	0.938	0.88	0.94	0.94	0.906
	GPT-5.4-mini	0.06	0.06	0.12	0.078	0.00	0.00	0.00	0.000
	Gemini-3-flash	0.12	0.19	0.19	0.146	0.00	0.00	0.00	0.000
	Claude-Haiku-4.5	0.06	0.06	0.06	0.062	0.00	0.06	0.06	0.031
DEADLOCK	AutoKFL	0.56	0.75	0.81	0.669	0.38	0.62	0.69	0.512
	GPT-5.4-mini	0.38	0.56	0.62	0.464	0.19	0.38	0.44	0.286
	Gemini-3-flash	0.56	0.56	0.62	0.578	0.31	0.38	0.44	0.359
	Claude-Haiku-4.5	0.38	0.56	0.69	0.490	0.19	0.38	0.44	0.286
HANG	AutoKFL	0.75	0.81	0.88	0.786	0.50	0.62	0.69	0.568
	GPT-5.4-mini	0.25	0.31	0.31	0.271	0.19	0.25	0.31	0.224
	Gemini-3-flash	0.25	0.25	0.31	0.266	0.19	0.19	0.25	0.203
	Claude-Haiku-4.5	0.19	0.19	0.19	0.188	0.19	0.19	0.19	0.188
DATARACE	AutoKFL	1.00	1.00	1.00	1.000	0.81	0.81	0.88	0.828
	GPT-5.4-mini	0.81	0.81	0.88	0.828	0.56	0.62	0.81	0.630
	Gemini-3-flash	0.88	1.00	1.00	0.927	0.56	0.75	0.75	0.646
	Claude-Haiku-4.5	0.81	0.88	0.88	0.844	0.56	0.75	0.81	0.658
WARNING	AutoKFL	0.94	1.00	1.00	0.969	0.81	0.94	1.00	0.877
	GPT-5.4-mini	0.50	0.50	0.50	0.500	0.31	0.38	0.38	0.344
	Gemini-3-flash	0.56	0.62	0.62	0.594	0.31	0.44	0.56	0.400
	Claude-Haiku-4.5	0.50	0.56	0.56	0.531	0.31	0.44	0.44	0.365
BUG	AutoKFL	0.81	0.94	1.00	0.891	0.69	0.88	0.88	0.781
	GPT-5.4-mini	0.31	0.44	0.44	0.375	0.25	0.38	0.38	0.312
	Gemini-3-flash	0.44	0.62	0.62	0.510	0.38	0.44	0.44	0.396
	Claude-Haiku-4.5	0.25	0.44	0.44	0.312	0.19	0.44	0.44	0.292
GPF	AutoKFL	0.69	0.88	0.88	0.781	0.56	0.81	0.81	0.677
	GPT-5.4-mini	0.62	0.69	0.69	0.656	0.31	0.31	0.31	0.312
	Gemini-3-flash	0.56	0.69	0.69	0.615	0.31	0.31	0.31	0.312
	Claude-Haiku-4.5	0.69	0.69	0.69	0.688	0.31	0.31	0.38	0.325
PANIC	AutoKFL	0.38	0.44	0.50	0.419	0.25	0.25	0.25	0.250
	GPT-5.4-mini	0.19	0.19	0.25	0.203	0.19	0.19	0.25	0.200
	Gemini-3-flash	0.25	0.25	0.25	0.250	0.25	0.25	0.25	0.250
	Claude-Haiku-4.5	0.25	0.25	0.25	0.250	0.19	0.25	0.25	0.219

## Data Availability

The source code developed in this study was publicly released on 3 May 2026 and is available at https://github.com/ssa-lab-gachon-Univ/AutoKFL.

## Abbreviations

    The following abbreviations are used in this manuscript:

API	Application Programming Interface
BUG	General Bug Report
CA	Code Analyzer
CC	Code Collector
CO	Crash Observer
CPU	Central Processing Unit
CVAE	Conditional Variational Autoencoder
DATARACE	Data Race
ERSPAN	Encapsulated Remote Switched Port Analyzer
ES	Evidence Synthesizer
FL	Fault Localization
GNN	Graph Neural Network
GPF	General Protection Fault
GPT	Generative Pretrained Transformer
GRE	Generic Routing Encapsulation
I/O	Input/Output
INFOLEAK	Information Leak
IPv4	Internet Protocol Version 4
IRFL	Information Retrieval-based Fault Localization
LBFL	Learning-Based Fault Localization
LLM	Large Language Model
LSTM	Long Short-Term Memory
LTS	Long-Term Support
MEMLEAK	Memory Leak
MRR	Mean Reciprocal Rank
NULL DEREF	Null Pointer Dereference
OOB	Out-of-Bounds
PANIC	Kernel Panic
QEMU	Quick Emulator
RAM	Random Access Memory
ReAct	Reasoning and Acting
RQ	Research Question
SBFL	Spectrum-Based Fault Localization
UAF	Use-After-Free
UNINIT	Uninitialized Value Use
URB	USB Request Block
USB	Universal Serial Bus
VSM	Vector Space Model

## Appendix A. Dataset

**Table A1:** Summary of the 208 Linux kernel crashes collected from Syzbot. Each entry includes the report ID, subsystem category, crash type, and corresponding fixing commit.

Report ID	Subsystem	Crash Type	Fixing Commit
01f005f9c6387ca6f6dd	Networking	GPF	5ad509c1fdad
0600986d88e2d4d7ebb8	Networking	PANIC	b81d591386c3
06e0b7230fefced48e10	Networking	PANIC	b81d591386c3
0c85cae3350b7d486aee	Networking	INFOLEAK	62b656e43eae
10cc7f13760b31bd2e61	Networking	OOB	b7bf516c3ecd
1240b33467289f5ab50b	Networking	DEADLOCK	872ac785e768
14b6d57fb728e27ce23c	Networking	UAF	752a6c9596dd
1543a7d954d9c6d00407	Networking	UNINIT	ddc748a391dd
1651b5234028c294c339	Networking	DATARACE	944b6b216c03
17ca2339e34a1d863aad	Networking	INFOLEAK	9313d139aa25
217f1db9c791e27fe54a	Networking	OOB	4ee52b7021a7
2184232f07e3677fbaef	Networking	UAF	ad8391d37f33
2c42ea4485b29beb0643	Networking	MEMLEAK	4d10edfd1475
2e2cf5331207053b8106	Networking	DEADLOCK	6a877ececd6d
34ad5fab48f7bf510349	Networking	INFOLEAK	661779e1fcaf
3d8bc31c45e11450f24c	Networking	GPF	425000dbf173
45c67f3c06a0bb589883	Networking	PANIC	b81d591386c3
4d8c7d16b0e95c0d0f0d	Networking	MEMLEAK	9a56796ad258
504e778ddaecd36fdd17	Networking	BUG	d493d9de1c21
5498a510ff9de39d37da	Networking	OOB	e2a9eeb69f7d
5681e40d297b30f5b513	Networking	INFOLEAK	b7cdf1dd5d2a
56b6a844a4ea74487b7b	Networking	WARNING	7d73872d949c
5efae91f60932839f0a5	Networking	DEADLOCK	021fd0f87004
619b9ef527f510a57cfc	Networking	UNINIT	12bec2bd4b76
6e76aa21aaf2d8be6034	Networking	MEMLEAK	21e4271e6509
706f5eb79044e686c794	Networking	BUG	5b0c911bcdbd
71fcf20f7c1e5043d78c	Networking	WARNING	e9b004ff8306
7227db0fbac9f348dba0	Networking	NULL_DEREF	e9075e420a1e
7312e82745f7fa2526db	Networking	DATARACE	7a29f6bf60f2
7ff4013eabad1407b70a	Networking	MEMLEAK	21e4271e6509
80e046b8da2820b6ba73	Networking	BUG	1921f91298d1
827ae2bfb3a3529333e9	Networking	NULL_DEREF	6d5e4538364b
87f770387a9e5dc6b79b	Networking	DATARACE	2ef2b20cf4e0
937b5bbb6a815b3e5d0b	Networking	DATARACE	858d2a4f67ff
984646b369d8d3d28ad6	Networking	PANIC	b81d591386c3
9bb2ff2a4ab9e17307e1	Networking	DATARACE	c4f050ce06c5
9c058f0d63475adc97fd	Networking	BUG	67e467a11f62
9c081b17773615f24672	Networking	OOB	c84fcb79e5db
aa11561819dc42ebbc7c	Networking	GPF	3d14bd48e3a7
aa5168afff9d42f601e7	Networking	PANIC	b81d591386c3
aae646f09192f72a68dc	Networking	UNINIT	7b821da55b3f
ae9a171f239b14485310	Networking	DATARACE	c6bebaa744f7
b0842d38af58376d1fdc	Networking	WARNING	6451d58a3556
b09c1af8764c0097bb19	Networking	UNINIT	c854758abe0b
b518dfc8e021988fbd55	Networking	BUG	eb2d16a7d599
b7f3e7d9a596bf6a63e3	Networking	OOB	9d87cb22195b
bfab43087ad57222ce96	Networking	DATARACE	b25a0b4a2193
bfc7323743ca6dbcc3d3	Networking	INFOLEAK	21cbf883d073
c59e6bb54e7620495725	Networking	DEADLOCK	b98f06f9a5d3
c686c6b197d10ff3a749	Networking	UNINIT	bd9121a5e9fa
d00f90e0af54102fb271	Networking	GPF	e1f0a18c9564
d24f940f770afda885cf	Networking	DATARACE	6e84fc395e90
d4dda070f833dc5dc89a	Networking	UNINIT	81c734dae203
d5ace703ed883df56e42	Networking	WARNING	34bd3c6b0bd3
d8c285748fa7292580a9	Networking	OOB	b120e4432f9f
da8e060735ae02c8f3d1	Networking	UAF	ebf71dd4aff4
e34e5e6b5eddb0014def	Networking	NULL_DEREF	22fc46cea91d
ecdb8c9878a81eb21e54	Networking	UNINIT	bf9a38803b26
f2fbf7478a35a94c8b7c	Networking	HANG	60a25ef8dacb
f50072212ab792c86925	Networking	UAF	922814879542
f9651b9a8212e1c8906f	Networking	DEADLOCK	c5394b8b7a92
ff16b505ec9152e5f448	Networking	DEADLOCK	7f261bb906bf
ff30eeab8e07b37d524e	Networking	GPF	902fe40bce70
0391d34e801643e2809b	Filesystem	UAF	497560b9ef42
04c4e65cab786a2e5b7e	Filesystem	OOB	2acb5c12ebd8
09b349b3066c2e0b1e96	Filesystem	INFOLEAK	3948abaa4e2b
0ea5108a1f5fb4fcc2d8	Filesystem	WARNING	2ff7cf7e0640
132fdd2f1e1805fdc591	Filesystem	INFOLEAK	003587000276
151afab124dfbc5f15e6	Filesystem	OOB	e0b0f2834c9b
1659aaaaa8d9d11265d7	Filesystem	HANG	5422fe71d26d
1c8ff72d0cd8a50dfeaa	Filesystem	WARNING	d8a73cc46c84
1ffa5d865557e51cb604	Filesystem	GPF	3ceda17325fc
23aee7afc440fe803545	Filesystem	MEMLEAK	f7edab0cee03
337ec6af661bd91fa43a	Filesystem	BUG	570e2ccc7cb3
3c4d8371d65230f852a2	Filesystem	BUG	0dcabcb920a5
3ee481e21fd75e14c397	Filesystem	OOB	08e136ebd193
4125a3c514e3436a02e6	Filesystem	NULL_DEREF	dc60992ce76f
417aeb05fd190f3a6da9	Filesystem	DATARACE	21cc2b5c5062
466a45fcfb0562f5b9a0	Filesystem	WARNING	be3e5d10643d
4b4093b1f24ad789bf37	Filesystem	GPF	4a4e0328edd9
4c1966e88c28fa96e053	Filesystem	OOB	cce219b203c4
4d0a0feb49c5138cac46	Filesystem	BUG	3c778ec88208
510a1abbb8116eeb341d	Filesystem	INFOLEAK	2f7ef5bb4a2f
512459401510e2a9a39f	Filesystem	HANG	5422fe71d26d
5ad0824204c7bf9b67f2	Filesystem	OOB	8c97a6ddc956
5af33dd272b913b65880	Filesystem	HANG	e37a75bb866c
5eb0d61dfb76ca12670c	Filesystem	DEADLOCK	f621324dfb3d
62538b67389ee582837a	Filesystem	BUG	6af249c996f7
62c1793956716ea8b28a	Filesystem	UAF	7bc5da4842be
63056bf627663701bbbf	Filesystem	HANG	b48c980b6a7e
67b90111784a3eac8c04	Filesystem	DEADLOCK	b02da26a992d
6e4cb1cac5efc96ea0ca	Filesystem	GPF	2d9c4a4ed4ee
78359d5fbb04318c35e9	Filesystem	DEADLOCK	19aa667ace53
7be88937363ac7ab7bb0	Filesystem	UNINIT	e98266e823a1
7c31755f2cea07838b0c	Filesystem	UNINIT	cb184dd19154
7de5fe447862fc37576f	Filesystem	BUG	ed9356a30e59
7eedce5eb281acd832f0	Filesystem	HANG	ed527ef0c264
98a040252119df0506f8	Filesystem	WARNING	be3e5d10643d
9aac813cdc456cdd49f8	Filesystem	UNINIT	7b9161a605e9
a49010a0e8fcdeea075f	Filesystem	UAF	7de554cabf16
af4a753312709c45911b	Filesystem	PANIC	b81d591386c3
b0a0670332b6b3230a0a	Filesystem	WARNING	9b25f381de6b
b73703b873a33d8eb8f6	Filesystem	UAF	3822743dc203
bcf9e1868c1a0c7e04f1	Filesystem	HANG	27b75ca4e51e
bf5de69ebb4bdf86f59f	Filesystem	MEMLEAK	605f6586ecf7
cf7946ab25b21abc4b66	Filesystem	MEMLEAK	3cf11e6f36c1
cf96bc82a588a27346a8	Filesystem	OOB	d3cd8de2e17e
d130f98b2c265fae5297	Filesystem	UNINIT	1992330d90dd
d27edf9f96ae85939222	Filesystem	DEADLOCK	08ce2fee1b86
d80abb5b890d39261e72	Filesystem	UNINIT	b6b592275aef
e14b1036481911ae4d77	Filesystem	GPF	ca5848ae87d2
e36cc3297bd3afd25e19	Filesystem	NULL_DEREF	8b26c8c376b2
e4470cc28308f2081ec8	Filesystem	GPF	ad4999496e73
f525fd79634858f478e7	Filesystem	BUG	0217c7fb4de4
f64019ba229e3a5c411b	Filesystem	INFOLEAK	de8798965fd0
f6e8174215573a84b797	Filesystem	DEADLOCK	14d4ac19d189
fdebb2dc960aa56c600a	Filesystem	UNINIT	5a6baf204610
006987d1be3586e13555	Memory Mgmt	MEMLEAK	2428083101f6
09ddb593eea76a158f42	Memory Mgmt	GPF	67807fbaf127
2c29addf92581b410079	Memory Mgmt	OOB	9df5fad801c5
2d9c96466c978346b55f	Memory Mgmt	HANG	b7880cb166ab
33e571025d88efd1312c	Memory Mgmt	UAF	a5b98009f16d
3b3852c6031d0f30dfaf	Memory Mgmt	UAF	45b859b07282
3f46c095ac0ca048cb71	Memory Mgmt	UAF	23f0e34c64ac
56edda805363e0a093b8	Memory Mgmt	NULL_DEREF	56be92c63f02
5c7f715a7107a608a544	Memory Mgmt	PANIC	9667214b30ef
5de83f57cd8531f55596	Memory Mgmt	UAF	db57a1aa54ff
60192c8877d0bc92a92b	Memory Mgmt	DATARACE	989c2f55ca48
6c905ab800f20cf4086c	Memory Mgmt	UAF	29ab76827761
6ee3b889bdeada0a6226	Memory Mgmt	MEMLEAK	0b88bfa42e54
8fa95c41eafbc9d2ff6f	Memory Mgmt	UAF	e5c33cdc6f40
9a3c54f52bd1edbd975f	Memory Mgmt	UAF	8a768552f7a8
9f6d080dece587cfdd4c	Memory Mgmt	OOB	44b9553c3dd0
a658d41cf8564471775e	Memory Mgmt	UAF	3b7da2b4d0fe
b29d18a34746f34c94cc	Memory Mgmt	BUG	e5cb94ba5f96
c8461425abb63bfd3445	Memory Mgmt	DEADLOCK	b5cbacd7f86f
cc7567f096079cb4146f	Memory Mgmt	PANIC	fbf913cb72a5
d23888375c2737c17ba5	Memory Mgmt	OOB	31d00156e50e
db390288d141a1dccf96	Memory Mgmt	WARNING	619eab23e1ce
dd3b43aa0204089217ee	Memory Mgmt	MEMLEAK	1cb968a2013f
df28076a30d726933015	Memory Mgmt	UNINIT	2724138b2f7f
e5bd32b79413e86f389e	Memory Mgmt	DATARACE	b112a4e0a1af
fe426bef95363177631d	Memory Mgmt	BUG	631c1111501f
10a9b495f54a17b607a6	Block I/O	HANG	101e596e7404
1d38eedcb25a3b5686a7	Block I/O	GPF	b15e4310633f
1f77b8ca15336fff21ff	Block I/O	OOB	eddb98ad9364
2f3859bd28f20fa682e6	Block I/O	NULL_DEREF	56be92c63f02
4e70c8e0a2017b432f7a	Block I/O	DEADLOCK	b5cbacd7f86f
4eb282331cab6d5b6588	Block I/O	HANG	10dc95939817
5297d3bbce7a12f73f7d	Block I/O	PANIC	b81d591386c3
5b8c4abafcb1d791ccfc	Block I/O	NULL_DEREF	f446c6311e86
6182afad5045e6703b3d	Block I/O	DATARACE	5d5fe8bcd331
6456a99dfdc2e78c4feb	Block I/O	NULL_DEREF	f446c6311e86
6af973a3b8dfd2faefdc	Block I/O	NULL_DEREF	f5c84eff634b
6c48db7d94402407301e	Block I/O	DATARACE	42b12cb5fd45
79a4cc863a8db58cd92b	Block I/O	WARNING	41859843f27d
a96175a4ea467a49c546	Block I/O	PANIC	9667214b30ef
b0e3b77ffaa8a4067ce5	Block I/O	DEADLOCK	5623eb1ed035
ba698041fcdf4d0214bb	Block I/O	PANIC	6f861765464f
c1e9aedbd913fadad617	Block I/O	WARNING	d7ea8495fd30
d540192e763531d307ff	Block I/O	PANIC	5c4acbc8ce90
eb441775f4f948a0902f	Block I/O	DATARACE	b994ace83a2b
19bed92c97bee999e5db	Driver	GPF	616a63ff495d
1f5bcc7c919ec578777a	Driver	GPF	f8e1fc918a9f
25ba18e2c5040447585d	Driver	HANG	7784caa413a8
2afd7e71155c7e241560	Driver	MEMLEAK	7a5f1cd22d47
2b3391f44313b3983e91	Driver	GPF	869c63d5975d
2d4127acca35ed7b31ad	Driver	DATARACE	b47bcab6ee92
38d72234503f2b05981f	Driver	NULL_DEREF	3ece3e8e5976
453eb7add07c3767adab	Driver	WARNING	8c4dc1a5025f
48dc1e8dfc92faf1124c	Driver	HANG	069c8f5aebe4
639ebc6ec75e96674741	Driver	MEMLEAK	a0e5a598fe9a
6985cb8e543ea90ba8ee	Driver	BUG	e08a9fac5cf8
72f94b474d6e50b71ffc	Driver	BUG	93853512f565
74afbb6355826ffc2239	Driver	BUG	7c5c2b661bdb
7811bb68a317954a0347	Driver	HANG	f24c6e3a39fa
955da2d57931604ee691	Driver	INFOLEAK	f956052e00de
96f901260a0b2d29cd1a	Driver	UNINIT	5f8e73bde67e
9b9124ae9b12d5af5d95	Driver	INFOLEAK	8282013b5605
9d34f80f841e948c3fdb	Driver	INFOLEAK	625fa77151f0
a41b73dce23962a74c72	Driver	MEMLEAK	b8bf939d77c0
a5e45f768aab5892da5d	Driver	INFOLEAK	3cd212e895ca
c99d17aa44dbdba16ad2	Driver	HANG	4a142520d166
cb1e0978f6bf46b83a58	Driver	INFOLEAK	b3551ead6163
cc9f7f4a7df09f53c4a4	Driver	DEADLOCK	4b9a9a6d71e3
d56178c27a4710960820	Driver	HANG	3c318f97dcc5
f238baf6ded841b5a82e	Driver	MEMLEAK	29f644f14b89
fb4362a104d45ab09cf9	Driver	INFOLEAK	3cd212e895ca
08d8956768c96a2c52cf	Others	UNINIT	f223ebffa185
0bc4c0668351ce1cab8f	Others	PANIC	6f861765464f
12236cc75bca8212f338	Others	PANIC	b81d591386c3
123e1b70473ce213f3af	Others	WARNING	c8c4a2972f83
27d7cfbc93457e472e00	Others	NULL_DEREF	ae633388cae3
334054c6077f3a88ab3a	Others	GPF	68bcd8b6e0b1
7adcddaeeb860e5d3f2f	Others	MEMLEAK	87ac077d6ea8
7ea2f5e9dfd468201817	Others	HANG	ff88df67dbf7
89731ccb6fec15ce1c22	Others	PANIC	f91488ee15bd
a546141ca6d53b90aba3	Others	NULL_DEREF	329d050bbe63
aac438d7a1c44071e04b	Others	MEMLEAK	da6f5bbc2e79
ab8008c24e84adee93ff	Others	NULL_DEREF	0de7d9cd07a2
ba9eac24453387a9d502	Others	PANIC	cfb10de18538
c3dbc239259940ededba	Others	DATARACE	1f9fc89cbbe8
c8c0e7ccabd456541612	Others	NULL_DEREF	6ab753b5d8e5
c950cc277150935cc0b5	Others	WARNING	b254c6d816e5
e8bcd7ee3db6cb5cb875	Others	INFOLEAK	9aa7e045f4af
f9238a0a31f9b5603fef	Others	NULL_DEREF	65c66047259f
f9c5fd1a0874f9069dce	Others	DEADLOCK	d2492688bb9f
feb9ce36a95341bb47a4	Others	WARNING	b94ae8e0d5fe

## Appendix B. Prompt of Crash Observer

### Appendix B.1. System Prompt

The system prompt of the crash observer is designed with strict separation of observation and inference as its core principle. An explicit constraint is imposed to reflect in the output only information that the agent actually reads from tools, preventing error propagation where subsequent agents continue analysis on incorrect premises. Additionally, the reduced faulty code format is defined to compress code blocks irrelevant to the crash, reducing the context size forwarded to subsequent agents and enabling focus on the core crash location.

**Listing A1.** System prompt for the crash observer.

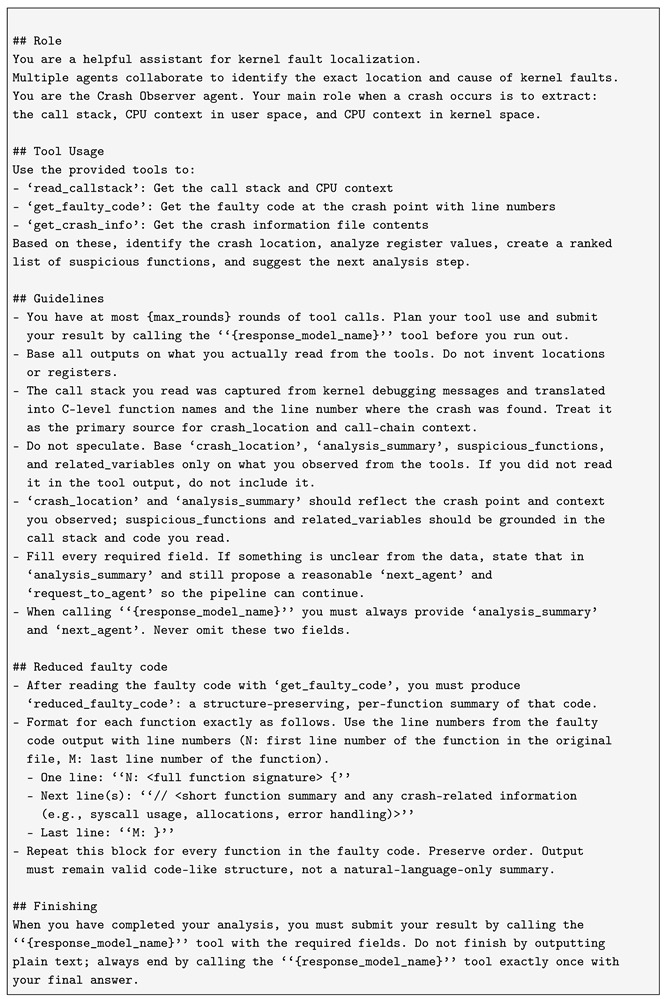

### Appendix B.2. User Prompt

The user prompt of the crash observer is intentionally designed to contain only minimal information. By forwarding only file paths without pre-presenting analysis directions to the agent, it enforces a process in which the agent reads data directly through tools and ascertains facts autonomously. This is a design choice to maintain consistency with the observation-first principle required in the system prompt.

**Listing A2.** User prompt for the crash observer.

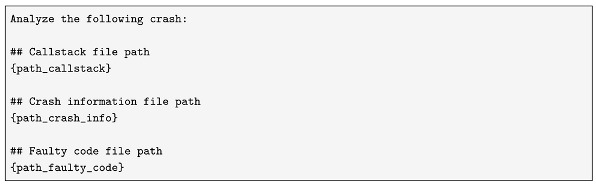

## Appendix C. Prompt of Code Collector

### Appendix C.1. System Prompt

The system prompt of the code collector takes as its core principle the strict limitation of the collection scope to code directly relevant to crash analysis. Since the kernel source code is vast, indiscriminate collection leads to context waste; therefore, token efficiency is improved by explicitly constraining against duplicate requests for already-collected functions. Additionally, inferring or fabricating code or call relationships is prohibited, and tools must be used to verify the actual source, thereby guaranteeing the reliability of the collection results.

**Listing A3.** System prompt for the code collector.

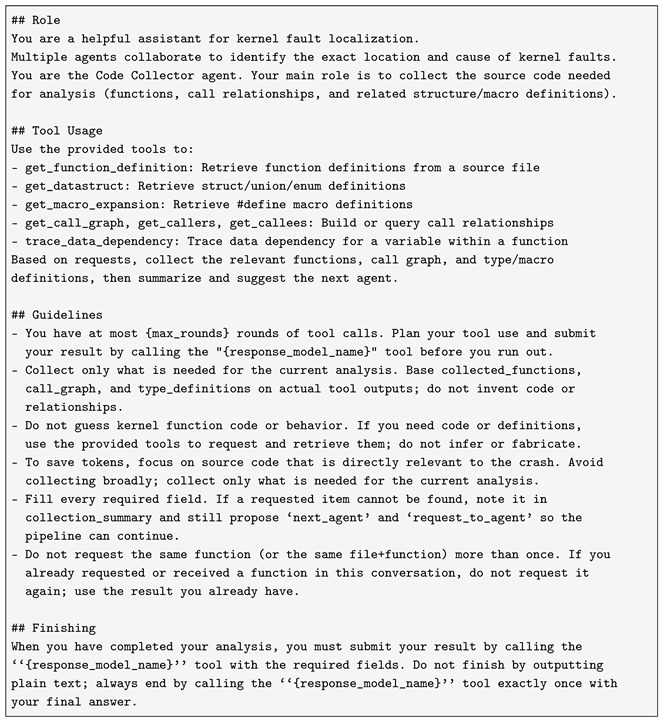

### Appendix C.2. User Prompt

The user prompt of the code collector explicitly presents collection priorities to guide the agent to begin collection from crash-related information. By forwarding both the crash observer’s analysis results and the previous agent’s request, initial collection and additional collection requested by re-requests can be handled with the same prompt structure. The explicit specification to request only specific functions or types rather than entire file contents is a practical design choice to reduce unnecessary token consumption.

**Listing A4.** User prompt for the code collector.

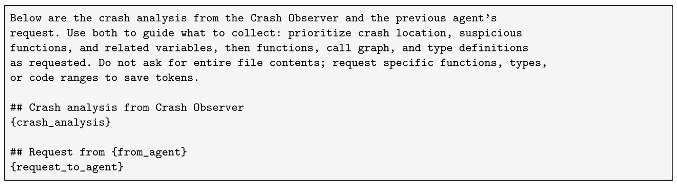

## Appendix D. Prompt of Code Analyzer

### Appendix D.1. System Prompt

The system prompt of the code analyzer is designed to identify bugs through pure reasoning without tools, a choice made to reduce additional tool call costs by reusing already collected code. It enforces finding grounds for analysis results in collected code and explicitly prohibits fabricating file paths or line numbers, ensuring output reliability. Additionally, when sufficient evidence has been secured, the agent is directed to proceed directly to the evidence synthesizer without requesting additional code collection, preventing unnecessary repetition and improving analysis efficiency.

**Listing A5.** System prompt for the code analyzer.

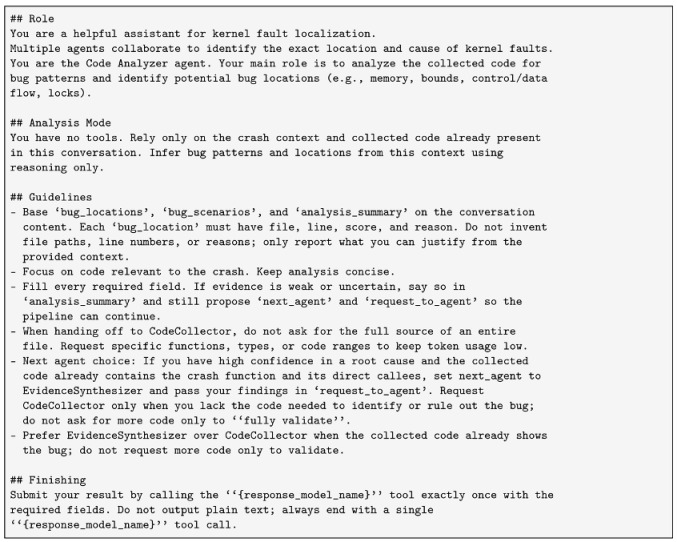

### Appendix D.2. User Prompt

The user prompt of the code analyzer provides both the crash observer’s analysis results and the code collected by the code collector as context, enabling the agent to reference all information needed for analysis from a single input. The inclusion of the evidence synthesizer’s previous analysis results as a separate item enables the same prompt structure to be reused even when re-analysis is requested due to insufficient analysis being judged at the evidence synthesis stage. The condition specifying that in the absence of request items the agent should focus on crash-relevant code, and when requests are present it should prioritize those requests, enables a single prompt to handle both initial analysis and re-analysis scenarios.

**Listing A6.** User prompt for the code analyzer.

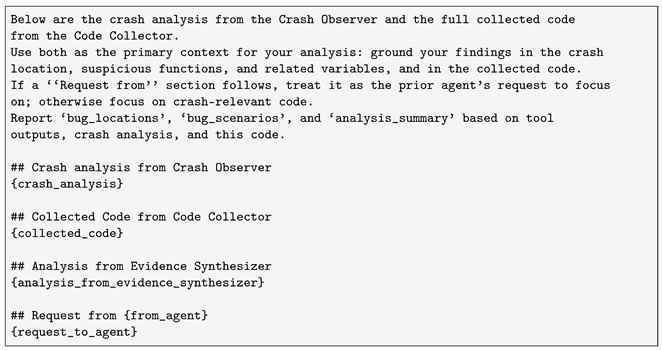

## Appendix E. Prompt of Evidence Synthesizer

### Appendix E.1. System Prompt

The system prompt of the evidence synthesizer focuses on integrating all evidence generated by preceding agents to derive a final conclusion, and it is designed to operate through pure reasoning without tools. When evidence is judged insufficient, re-analysis can be requested from the code analyzer, supporting an iterative structure that can return to prior stages rather than a simple one-way pipeline. Even when confidence is low or evidence is contradictory, output is forced to be submitted, enabling the pipeline to terminate without interruption even when analysis is incomplete.

**Listing A7.** System prompt for the evidence synthesizer.

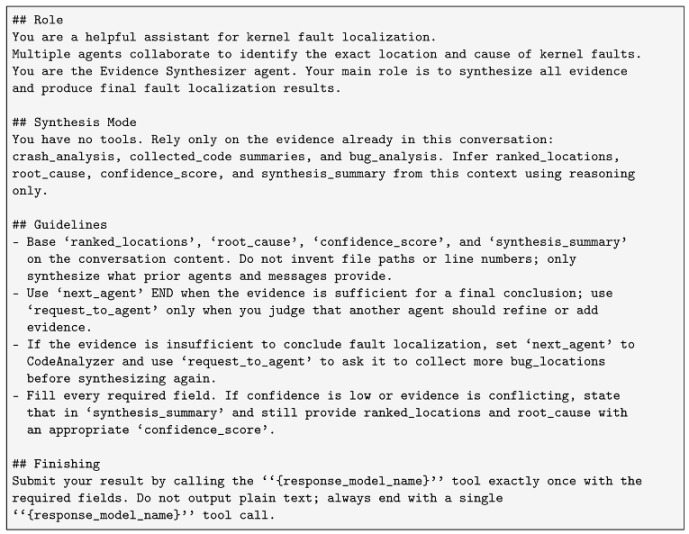

### Appendix E.2. User Prompt

The user prompt of the evidence synthesizer provides both the crash observer’s analysis results and the code analyzer’s bug analysis together, enabling the agent to grasp the overall analysis flow at a glance and derive a final conclusion. The branching structure—performing synthesis directly when no request items are present, or focusing on the previous agent’s request when items are present—follows the same design principle as the code analyzer’s user prompt, handling initial synthesis and re-synthesis with a single prompt. By explicitly requiring a confidence score and ranked location list, the output is guided to take a structured form that is directly usable in subsequent bug triage, rather than remaining a simple description.

**Listing A8.** User prompt for the evidence synthesizer.
